# Characteristics, care and support needs of older Victorians requiring a government‐funded Home Care Package: An observational study

**DOI:** 10.1111/ajag.13400

**Published:** 2025-01-12

**Authors:** Rachel McKittrick, Liliana Orellana, Elizabeth Manias, Martin Hensher, Alison M. Hutchinson

**Affiliations:** ^1^ School of Nursing and Midwifery, Faculty of Health Deakin University Burwood Victoria Australia; ^2^ Biostatistics Unit, Faculty of Health Deakin University Geelong Victoria Australia; ^3^ Monash Nursing and Midwifery, Faculty of Medicine, Nursing and Health Science Monash University Clayton Victoria Australia; ^4^ Menzies Institute for Medical Research University of Tasmania Hobart Tasmania Australia; ^5^ School of Nursing and Midwifery, Faculty of Health Deakin University Geelong Victoria Australia; ^6^ Centre for Quality and Patient Safety Research, Institute for Health Transformation Deakin University Geelong Victoria Australia; ^7^ Barwon Health Geelong Victoria Australia

**Keywords:** aged, health services for the aged, health workforce, home care services, needs assessment

## Abstract

**Objectives:**

To describe sociodemographic characteristics and comprehensive day‐to‐day care and support needs of older Victorians requiring government‐funded home‐based aged‐care, and to explore associations between vulnerability factors and complexity indicators in this population.

**Methods:**

A population‐based observational study was conducted using de‐identified, routinely collected aged‐care assessment data for Victorians approved for a Home Care Package (HCP) between January 2019 and June 2022.

**Results:**

The study population (*n* = 94,975 individuals), approved for one of four HCP levels (Levels 1 (5%), 2 (38%), 3 (34%) or 4 (24%)), was aged 82 years on average (SD 7.6), commonly born outside Australia (48%), with people of higher socio‐economic status more likely to be approved for a high‐level HCP. Advanced care and support needs such as for showering (53%) and dressing (43%) were common, with higher overall needs when cognitive and behavioural concerns such as short‐term memory loss (75%) or agitation (21%) were present. 79% reported at least one vulnerability factor such as being socially isolated, culturally and linguistically diverse, or Aboriginal and Torres Strait Islander peoples, and 49% reported at least one complexity indicator. The three most prevalent complexity indicators were significant cognitive changes (29%), self‐neglect (17%) and emotional/mental health issues (11%), which were generally positively associated with vulnerability factors.

**Conclusions:**

This population‐based study provides evidence of the diverse sociodemographics, and often advanced day‐to‐day mobility, functional, physical, cognitive, behavioural, psychological and psychosocial care and support needs of people requiring home‐based aged‐care. It also highlights the multifaceted complexities within this population. Results could inform home care service‐delivery models and workforce skill‐mix requirements to efficiently and safely meet these needs.

**Policy Impact**

A comprehensive understanding of the characteristics, care and support needs of all Victorians needing a Home Care Package is valuable for aged care policymakers when developing home‐based aged care service delivery models and when undertaking workforce skill‐mix planning.

**Practice Impact**

The findings from this study are useful for aged care providers as they consider their home care service models for clients and the skills and skill‐mix required across their workforce to safely and holistically meet the diverse, often high and complex care and support needs of their clients.

## 1 INTRODUCTION

Australia's ageing population is set to place exceptional demand on the home‐based aged‐care system in the coming decades.^1^ This demand is in part due to most older people preferring to receive aged‐care in their own homes rather than residential aged‐care facilities.^2^ Other system pressures include increasing expectations regarding the quality of aged‐care, considerable workforce shortages and escalating care provision costs. Combined, these supply and demand factors are leading to significant increases in government spending on home‐based aged‐care.^3^ Given this situation, well‐targeted utilisation of available government funding and human resources is vital. To do this, home‐based aged‐care service‐delivery model design and workforce planning must be guided by a detailed understanding of the population's needs.^4^ Without this understanding, there is a risk service‐delivery models will not be structured to comprehensively deliver the care and support required to meet those needs. Further, the workforce may lack the skills and skill‐mix to deliver safe, quality care, thereby leading to ineffective and inefficient use of limited resources.^5^

In Australia, some research has been conducted to understand certain aspects of the needs of Australians requiring home‐based aged‐care. This includes government‐funded studies assessing service‐type utilisation across current home‐based aged‐care programs.^6^, ^7^ However, understanding service‐type utilisation is different to understanding the underlying needs of this population group. Research about the underlying needs of older people requiring home‐based aged‐care has focused on people with specific conditions such as dementia,^8^, ^9^, ^10^ particular population groups such as veterans,^11^ or certain issues such as social isolation,^12^ falls,^13^ multi‐morbidity and frailty^14^ and hospitalisations.^15^ Some of these studies have reported sociodemographic characteristics of Australians requiring or receiving home‐based aged‐care.^14^, ^16^ Australians receiving home‐based aged‐care are predominantly in their 70s and 80s, more likely to be female, and approximately one‐third were born outside Australia.^17^ These studies provide useful perspectives about sub‐population groups and various aspects of the needs of people requiring home‐based aged‐care. Yet, as a recent scoping review showed [unpublished data], there is little research focused on providing insights into the holistic range of underlying mobility, functional, physical, cognitive, behavioural, psychological day‐to‐day care and support needs of this population; nor is there any research providing information about the prevalence of these needs across the population. Also, few studies have focused on the overall complexity of individuals' care and support needs that can challenge service‐delivery.^18^ Seeking to understand this complexity is essential to enhance understanding of the multifaceted nature of care and support needs, alongside understanding the types of day‐to‐day care and support needs commonly experienced by older people requiring home‐based aged‐care services. Some research has been conducted in Canada,^19^ the United States^20^ and New Zealand^21^ about the comprehensive needs of home‐based older people, finding populations accessing home‐care programs have an extensive range of multidimensional and complex needs. The study authors argue that evidence‐based understanding of such needs is essential for optimal conceptualisation of care models at the system, service and clinical levels. Subsequent benefits include services being more aligned with needs, resulting in lower unmet needs for the population, and improved outcomes such as better quality of life, delayed entry to residential care and fewer hospital admissions.^19^

Presently, Australian home‐based aged‐care is delivered through two main programs. The Commonwealth Home Support Programme (CHSP) provides ‘entry‐level’ support, and the Home Care Package (HCP) program assists those with increasingly complex care and support needs.^22^ In the context of population ageing, increasing numbers of people are developing complex care and support needs, hence requiring HCP‐type support. Improved understanding of the needs of this vulnerable population group is therefore a priority. Accordingly, the context for this study is the HCP program. As depicted in Table 1, the HCP program consists of four levels, where Level‐1 supports people with ‘basic’ needs, through to Level‐4 that supports people with ‘high’ needs. At the time of this research, the CHSP and HCP programs were undergoing reform to create a combined ‘support‐at‐home’ program.^23^

**TABLE 1:** Home Care Package (HCP) program care levels.^24^

HCP level^a^, ^b^	Level of care needs
Level 1	Basic‐level care and support needs—generally require infrequent assistance and management, and/or intermittent intervention
Level 2	Low‐level care and support needs—generally require regular assistance and management, and/or minimal intervention
Level 3	Intermediate‐level care and support needs—generally require frequent assistance and management, and/or regular intervention
Level 4	High‐level care and support needs—generally require comprehensive assistance and management, and/or frequent intervention

^a^ Each HCP level equates to a prescribed Australian Government subsidy per year: Level 1 ($9026.45; Level 2 ($15,887.50); Level 3: $34,550.90; Level 4: $52,377.50), which is used to fund flexible and personalised direct care services that an older person requires to remain living safely at home. These services can range from cleaning and shopping assistance to personal care, nursing and allied health services for more complex needs, and assistive equipment and technology. Spending of these funds is set out in the *Home Care Packages Program Operational Manual—a guide for home care providers*.^25^

^b^ A person is eligible for a HCP when they have needs that can only be met by a coordinated package of care services, as set out in the *My Aged Care assessment manual—for Regional Assessment Services and Aged Care Assessment Teams*.^22^

Using a large aged‐care assessment dataset about Victorians eligible for a HCP, the aim of this study was to describe the range and prevalence of sociodemographic characteristics, and comprehensive day‐to‐day care and support needs of older people living in Victoria (a state of Australia), both across the total study population and by HCP approval level. A further aim was to explore associations between vulnerability factors (e.g. being socially isolated, culturally and linguistically diverse or Aboriginal and Torres Strait Islander peoples) and complexity indicators (e.g. experiencing significant cognitive changes, self‐neglect, emotional/mental health issues) in this population.

## 2 METHODS

### 2.1 Study design and data source

A population‐based observational study was conducted using the National Screening and Assessment Form (NSAF) dataset (2022 version). The dataset comprises information about an individual's care and support needs, routinely collected by aged‐care assessors in an electronic NSAF when assessing an individual's HCP eligibility.^22^ This routinely collected NSAF information for the Australian population is stored in the National Aged Care Data Clearinghouse under custodianship of the Australian Institute of Health and Welfare (AIHW). The AIHW provided access to the de‐identified NSAF‐dataset for the population of interest, for the purposes of this study. Approval to conduct this study was provided by Deakin University Human Research Ethics Committee (2022‐132).

**FIGURE 1:**
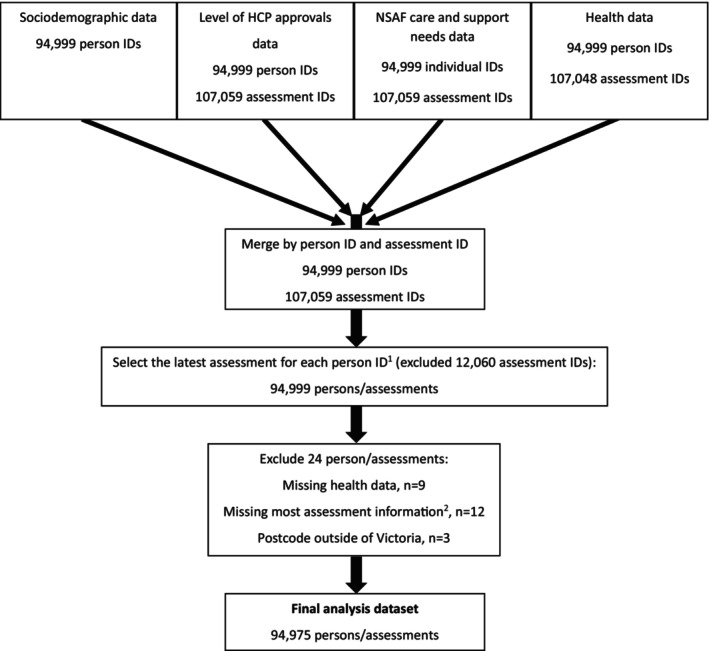
Flowchart of National Screening and Assessment Form (NSAF) data file merging and inclusion/exclusion decision. ID, identifier; NSAF, National Screening and Assessment Form. ^1^The latest assessment was retained for any individual who had undergone more than one assessment, as the most up‐to‐day reflection of an individual's needs. ^2^ >80% of the care and support needs variables were missing.

De‐identified data for 94,999 individuals across 107,059 assessments were obtained from the AIHW across four separate data files (one each for sociodemographics, level of HCP approval, care and support needs and health data; Figure 1), which were merged using person and assessment identifiers contained within each file. For people who had undergone more than one assessment, their latest assessment was retained as the most recent reflection of their needs. Twenty‐four individuals were excluded because their record contained no or limited assessment or health data, or they did not reside in Victoria. The final dataset included 94,975 individuals, each contributing one assessment (Figure 1), so analysis was conducted at the individual level.

### 2.2 Population of interest

The study included all individuals residing in Victoria, Australia, approved for HCP (any level) between January 2019 and June 2022.

### 2.3 Study variables

The NSAF variables included in this analysis were as follows: (1) *sociodemographic characteristics* (e.g. age, postcode, living‐situation); (2) *physical considerations*, including *activities‐of‐daily‐living* (e.g. walking, showering, bladder use) and *other care concerns* (e.g. with vision, speech, falls); (3) *cognitive/behavioural/psychological considerations* (e.g. short‐term memory loss, impaired‐judgement, anxiety); (4) *psychosocial considerations*, including *vulnerability factors* (e.g. socially isolated, culturally and linguistically diverse, Aboriginal and Torres Strait Islander peoples, carer‐sustainability concerns) and *complexity indicators* (e.g. significant cognitive changes, self‐neglect, emotional/mental health issues); (5) *health concerns* (i.e. health conditions); and (6) *health‐care use* (e.g. sees a GP regularly, taking medication, hospitalisations). The NSAF‐dataset also included *approved HCP‐level*. We further derived multi‐vulnerability, multi‐complexity and multi‐morbidity variables by counting the total number of vulnerabilities, complexities and health conditions per person. Appendix S1 defines the scope of all variables in the dataset.

Residential postcode for each individual within the dataset was used to define area‐level socio‐economic position using the Index‐of‐Relative‐Socio‐economic‐Advantage‐and‐Disadvantage (IRSAD). The IRSAD summarises information about the economic and social situations of people and households within postcodes. The lower the IRSAD postcode decile, the more relative disadvantage residents within those postcodes are likely to experience.^26^

Missing data were minimal. For sociodemographic variables, the overall percentage of missing data was 1% (three variables had approximately 2% missing data and the rest less than 1%). For care and support needs variables, the overall percentage of missing data was 0%.

### 2.4 Statistical analysis

Following cleaning, these data were analysed descriptively and reported as frequencies and percentages for the whole population and by approved HCP‐level. Due to the large sample size, all *p*‐values were highly significant and hence not informative; therefore, they are not reported. Associations between vulnerability factors and the three most commonly reported complexity indicators overall (from Table 3: significant cognitive changes, self‐neglect and emotional/mental health issues; see Appendix S1 for definitions) were explored. Association estimates have been reported as risk ratios with 95% confidence intervals (CI), with the vulnerability factor as the exposure. Data cleaning and analysis were performed using the Stata version‐18 software.

## 3 RESULTS

The results from descriptive analysis of the characteristics, care and support needs of this population are reported for the whole population and by approved HCP‐level in Tables 2 and 3 (select characteristics, care and support needs results only; see Appendix S2 for all characteristics, care and support needs results). Associations between vulnerability factors and complexity indicators are shown in Table 4.

**TABLE 2:** Prevalence of selected sociodemographic characteristics amongst people approved for a Home Care Package (HCP) between January 2019 and June 2022: for all and by HCP.

Approved HCP level	All	HCP L1	HCP L2	HCP L3	HCP L4
	*n* (%)	*n* (%)	*n* (%)	*n* (%)	*n* (%)
HCP approval rates	94,975 (100)	4883 (5)	35,723 (38)	32,031 (34)	22,338 (23.5)
Age in years—mean (SD)	82 (8)	80 (7)	82 (7)	82 (8)	83 (8)
Gender—females^a^	57,253 (60)	2810 (5)	22,231 (39)	19,450 (34)	12,762 (22)
Birth country^b^					
Australia	49,115 (52)	2650 (5)	18,851 (38)	16,735 (34)	10,879 (22)
Other	44,385 (47)	2161 (5)	16,238 (37)	14,842 (33)	11,144 (25)
Preferred language^c^					
English	72,297 (76)	3950 (6)	27,624 (38)	24,505 (34)	16,218 (22)
Greek	5037 (5)	229 (5)	1787 (36)	1639 (33)	1382 (27)
Italian	4867 (5)	136 (3)	1712 (35)	1585 (33)	1434 (30)
Other	10,822 (11)	487 (5)	3834 (35)	3651 (34)	2850 (26)
Living alone^d^	37,342 (39)	1481 (4)	15,115 (41)	13,051 (35)	7695 (21)
Geographical remoteness (MMM code) within Victoria^e^					
Metro	69,671 (73)	3470 (5)	25,701 (37)	23,469 (34)	17,031 (24)
Regional	6364 (7)	250 (4)	2486 (39)	2249 (35)	1379 (22)
Large rural	5984 (6)	387 (7)	2555 (43)	1928 (32)	1114 (19)
Medium rural	6395 (7)	416 (7)	2521 (39)	2146 (34)	1312 (21)
Small rural, remote and very remote	6516 (7)	360 (6)	2443 (38)	2225 (34)	1488 (23)
IRSAD (Victorian deciles)^f,^ *					
1st (least advantaged/most disadvantage)	14,041 (15)	598 (4)	5320 (38)	5022 (36)	3101 (22)
2nd	6758 (7)	324 (5)	2771 (41)	2345 (35)	1318 (20)
3rd	8163 (9)	401 (5)	3132 (38)	2769 (34)	1861 (23)
4th	6021 (6)	226 (4)	2148 (36)	2162 (36)	1485 (25)
5th	9126 (10)	370 (4)	3376 (37)	3317 (36)	2063 (23)
6th	5218 (6)	228 (4)	2008 (39)	1787 (34)	1195 (23)
7th	8595 (9)	448 (5)	3344 (39)	2828 (33)	1975 (23)
8th	12,231 (13)	944 (8)	4879 (40)	3811 (31)	2597 (21)
9th	14,071 (15)	965 (7)	5232 (37)	4525 (32)	3349 (24)
10th (most advantaged/least disadvantage)	10,701 (11)	379 (4)	3494 (33)	3449 (32)	3379 (32)

*Note*: Missing data: ^a^3 (<.001%); ^b^1475 (1.6%); ^c^1952 (2.1%); ^d^256 (.3%); ^e^45 (.1%); ^f^50 (.1%).

Abbreviations: IRSAD, Index of Relative Advantage and Disadvantage; MMM, Modified Monash Model.

* IRSAD has been calculated using the provided ‘postcode’ variable, as a measure, which summarises information about the economic and social conditions of people and households within areas, including both relative advantage and disadvantage measures, as set out by the Australian Bureau of Statistics.^26^

**TABLE 3:** Prevalence of selected physical, cognitive/behavioural/psychological care and psychosocial considerations, health concerns and healthcare use amongst people approved for a Home Care Package (HCP) between January 2019 and June 2022: for all and by HCP.

Approved HCP level	All	HCP L1	HCP L2	HCP L3	HCP L4
	*n* (%)	*n* (%)	*n* (%)	*n* (%)	*n* (%)
HCP approval rates	94,975 (100)	4883 (5)	35,723 (38)	32,031 (34)	22,338 (24)
Physical considerations: activities‐of‐daily‐living					
Mobility					
Walking					
Some assistance	54,190 (57)	1122 (2)	15,256 (28)	21,325 (39)	16,487 (30)
High level of assistance/unable	2008 (2)	6 (0)	109 (5)	236 (12)	1657 (83)
Transfers*					
Some assistance	37,052 (39)	508 (1)	8393 (23)	14,582 (39)	13,569 (37)
High level of assistance/unable	1072 (1)	2 (0)	35 (3)	74 (7)	961 (90)
Personal care					
Showering/bathing					
Some assistance	46,927 (49)	489 (1)	9348 (20)	20,301 (43)	16,789 (36)
High level of assistance/unable	3071 (3)	3 (0)	114 (4)	367 (12)	2587 (84)
Dressing					
Some assistance	38,654 (41)	448 (1)	6950 (18)	15,806 (41)	15,450 (40)
High level of assistance/unable	2485 (3)	1 (0)	69 (3)	229 (9)	2186 (88)
Toileting (bladder)					
Some assistance	25,667 (27)	342 (1)	4871 (19)	8963 (35)	11,491 (45)
High level of assistance/unable	1895 (2)	1 (0)	75 (4)	207 (11)	1612 (85)
Toileting (bowel)^a^					
Some assistance	17,824 (19)	142 (1)	2540 (14)	5708 (32)	9434 (53)
High level of assistance/unable	1354 (1.4)	1 (0)	50 (4)	96 (7)	1207 (89)
Medication management					
Some assistance	49,862 (53)	788 (2)	12,615 (25)	19,746 (40)	16,713 (34)
High level of assistance/unable	3951 (4)	8 (0)	274 (7)	931 (24)	2738 (69)
Eating/feeding					
Some assistance	16,432 (17)	77 (1)	1618 (10)	5087 (31)	9650 (59)
High level of assistance/unable	373 (0)	0 (0)	15 (4)	22 (6)	336 (90)
Physical considerations: other care concerns					
Poor vision^†^	61,077 (64)	2834 (5)	22,760 (37)	21,142 (35)	14,341 (24)
Poor hearing^†^	45,377 (48)	2018 (5)	16,506 (36)	15,640 (35)	11,213 (25)
Speech issues^†^	7221 (8)	132 (2)	1314 (18)	2475 (34)	3300 (46)
Swallowing problems^b^	12,771 (14)	307 (2)	3282 (26)	4481 (35)	4701 (37)
Communication difficulties^c^	35,415 (37)	772 (2)	9075 (26)	12,683 (36)	12,885 (36)
Food, fluid and/or weight concerns^a^	38,921 (41)	1059 (3)	11,692 (30)	14,156 (36)	12,014 (31)
Has difficulty sleeping^b^	51,944 (55)	2364 (5)	19,028 (37)	17,659 (34)	12,893 (25)
Falls/slips/trips in last 12 months^d^	56,066 (59)	1962 (4)	18,687 (33)	20,110 (36)	15,307 (27)
Experienced bodily pain last 4 weeks^d^	71,261 (75)	3751 (5)	27,514 (39)	24,216 (34)	15,780 (22)
Cognitive/behavioural/psychological considerations					
Specific cognitive care concerns					
Short‐term memory loss^a^					
Occasionally	44,076 (46)	2317 (5)	18,796 (43)	15,253 (35)	7710 (18)
Regularly	21,806 (23)	309 (1)	5117 (24)	8484 (39)	7896 (36)
Always	5588 (6)	23 (0)	492 (9)	1608 (29)	3465 (62)
Disorientation—time^d^					
Occasionally	17,694 (19)	329 (2)	4542 (26)	6906 (39)	5917 (33)
Regularly	7349 (8)	42 (1)	820 (11)	2462 (34)	4025 (55)
Always	1496 (2)	3 (0)	78 (5)	343 (23)	1072 (72)
Disorientation—place^d^					
Occasionally	10,101 (11)	89 (1)	1860 (18)	3770 (37)	4382 (43)
Regularly	3205 (3)	10 (0)	202 (6)	858 (27)	2135 (67)
Always	542 (1)	0 (0)	19 (4)	89 (16)	434 (80)
Disorientation—person^d^					
Occasionally	8945 (9)	95 (1)	1741 (20)	3119 (35)	3990 (45)
Regularly	2211 (2)	11 (1)	151 (7)	540 (24)	1509 (68)
Always	283 (0)	0 (0)	12 (4)	40 (14)	231 (82)
Specific behavioural care concerns					
Impaired judgement^a^					
Occasionally	16,807 (18)	212 (1)	3766 (22)	6641 (40)	6188 (37)
Regularly	4886 (5)	18 (0)	458 (9)	1445 (30)	2965 (61)
Always	634 (1)	1 (0)	29 (5)	94 (15)	510 (80)
Agitation^a^					
Occasionally	17,105 (18)	440 (3)	4469 (26)	6226 (36)	5970 (35)
Regularly	3147 (3)	57 (2)	525 (17)	1024 (33)	1541 (49)
Always	110 (0)	2 (2)	16 (15)	33 (30)	59 (54)
Apathy^a^					
Occasionally	12,894 (14)	344 (3)	3802 (30)	4794 (37)	3954 (31)
Regularly	4499 (4.7)	50 (1)	881 (20)	1676 (37)	1892 (42)
Always	448 (.5)	2 (1)	57 (13)	122 (27)	267 (60)
Confusion^a^					
Occasionally	22,525 (24)	352 (2)	5931 (26)	8935 (40)	7307 (32)
Regularly	7924 (8)	22 (0)	811 (10)	2555 (32)	4536 (57)
Always	1114 (1)	0 (0)	46 (4)	219 (20)	849 (76)
Specific psychological care concerns					
Insomnia^a^					
Occasionally	30,039 (32)	1489 (5)	11,336 (38)	10,138 (34)	7076 (24)
Regularly	27,651 (29)	1244 (5)	9959 (36)	9530 (35)	6918 (25)
Always	4875 (5)	227 (5)	1738 (36)	1541 (32)	1369 (28)
Depression^a^					
Occasionally	36,679 (39)	1482 (4)	13,465 (37)	12,889 (35)	8843 (24)
Regularly	12,413 (13)	335 (3)	3827 (31)	4732 (38)	3519 (28)
Always	1114 (1)	27 (2)	302 (27)	411 (37)	374 (34)
Anxiety^a^					
Occasionally	38,744 (41)	1774 (5)	14,375 (37)	13,347 (35)	9248 (24)
Regularly	14,950 (16)	476 (3)	4852 (33)	5458 (37)	4164 (28)
Always	1440 (2)	52 (4)	409 (28)	510 (35)	469 (33)
Psychosocial considerations					
Social support situation/carer status*					
Has a carer^a^	76,870 (81)	2687 (4)	26,059 (34)	27,526 (36)	20,598 (27)
Vulnerability factors^‡^					
Socially isolated	57,218 (60)	1796 (3)	20,375 (36)	20,646 (36)	14,401 (25)
Culturally and linguistically diverse	34,279 (36)	1453 (4)	12,253 (36)	11,561 (34)	9012 (26)
Carer‐sustainability concerns	20,236 (21)	442 (2)	5481 (27)	7175 (36)	7138 (35)
War veterans	1032 (1)	41 (4)	323 (31)	386 (37)	282 (27)
Aboriginal and Torres Strait Islanders	684 (1)	29 (4)	260 (38)	249 (36)	146 (21)
Asylum seeker or refugee	398 (0)	10 (3)	113 (28)	147 (37)	128 (32)
Gender diverse	127 (0)	5 (4)	44 (35)	33 (26)	45 (35)
Muti‐vulnerabilities^‡^					
0	19,873 (21)	2017 (10)	8647 (44)	5786 (29)	3423 (17)
1	42,529 (45)	2041 (5)	16,739 (39)	14,626 (34)	9123 (22)
2	26,430 (28)	743 (3)	8933 (34)	9347 (35)	7407 (28)
3+	6143 (7)	82 (1)	1404 (23)	2272 (37)	2385 (39)
Complexity indicators^‡^					
Significant cognitive changes	27,350 (29)	213 (1)	4734 (17)	10,194 (37)	12,209 (45)
Self‐neglect	16,168 (17)	148 (1)	2295 (14)	5856 (36)	7869 (49)
Emotional/mental health issues	10,182 (11)	173 (2)	2577 (25)	3981 (39)	3451 (34)
Financial disadvantage	5319 (6)	136 (3)	1566 (29)	2000 (38)	1617 (30)
Inadequate housing	1649 (2)	53 (3)	526 (32)	628 (38)	442 (27)
Risk of, suspected or confirmed abuse	1555 (2)	37 (2)	427 (28)	602 (39)	489 (32)
Drug and alcohol use likely to cause harm to self or others	1245 (1)	22 (2)	346 (28)	502 (40)	375 (30)
History of institutionalisation	742 (1)	18 (2)	182 (25)	281 (38)	261 (35)
Multi‐complexities^‡^					
0	53,314 (56)	4245 (8)	26,401 (50)	16,167 (30)	6501 (12)
1	25,312 (27)	511 (2)	6854 (27)	9942 (39)	8005 (32)
2	11,819 (12)	98 (1)	1857 (16)	4274 (36)	5590 (47)
3+	4530 (5)	29 (1)	611 (14)	1648 (36)	2242 (50)
Health concerns^§^					
Chronic diseases					
Musculoskeletal system diseases (including arthritis)	54,841 (58)	3083 (6)	22,382 (41)	18,366 (34)	11,010 (20)
Heart disease (includes IHD/past MI)	30,253 (32)	1357 (5)	11,255 (37)	10,568 (35)	7073 (23)
Mental health issues	25,630 (27)	933 (4)	8917 (35)	9397 (37)	6383 (25)
Diabetes (includes T1DM/T2DM)	24,854 (26)	1025 (4)	8839 (36)	8604 (35)	6386 (26)
Diagnosed dementia (all types)	11,438 (12)	100 (1)	1970 (17)	3946 (35)	5422 (47)
CCF	6123 (6)	158 (3)	1750 (29)	2321 (38)	1894 (31)
Parkinson's Disease	4618 (5)	77 (2)	1010 (22)	1711 (37)	1820 (39)
Other health‐related signs and symptoms					
Cognitive impairment (no recorded diagnosis of dementia)	13,450 (14)	238 (2)	3936 (27)	5661 (38)	5007 (34)
Falls	13,931 (15)	237 (2)	3974 (29)	5298 (38)	4422 (32)
Abnormal gait/mobility	12,664 (13)	208 (2)	3558 (28)	4889 (39)	4009 (32)
Incontinence (urinary or bowel)	9472 (10)	198 (2)	2544 (27)	3283 (35)	3447 (36)
Nutritional disorders (includes malnutrition)	3915 (4)	113 (3)	1233 (32)	1385 (35)	1184 (30)
Multi‐morbidity					
0	18 (0)	3 (17)	12 (67)	2 (11)	1 (6)
1–2	5691 (6)	540 (10)	2322 (41)	1681 (30)	1148 (20)
3+	89,266 (94)	4340 (5)	33,389 (37)	30,348 (34)	21,189 (24)
Health‐care use					
Hospitalisations^a^					
In past 3 months	38,639 (41)	1249 (3)	12,725 (33)	14,022 (36)	10,643 (28)
Currently in hospital	2667 (3)	24 (1)	642 (24)	823 (31)	1178 (44)

*Note*: Missing data: ^a^2 (<.001%); ^b^3 (<.001%); ^c^4 (<.001%); ^d^1 (<.001%).

Abbreviations: CCF, congestive heart failure; IHD, ischaemic heart disease; MI, myocardial infarction; T1DM, type 1 diabetes; T2DM, type 2 diabetes.

* Transfers refers to moving about in/getting in and out of bed, as well as getting on/off chairs and the toilet, and getting in/out of the shower/car.

^†^ A response is only recorded for vision/blindness, hearing/deafness and speech when this concern is applicable.

^‡^ Vulnerability factors and complexity indicators are only recorded where applicable and >1 factor/indicator may apply per person.

^§^ See Appendix S2 for all health conditions.

**TABLE 4:** Association between vulnerability factors and complexity indicators amongst people approved for a Home Care Package (HCP) between January 2019 and June 2022: for all and by HCP.

Vulnerabilities		Prevalence of the vulnerability in whole sample	Most commonly reported complexity indicators					
			Significant cognitive changes		Self‐neglect		Emotional/mental health issue	
			Risk within levels of vulnerability		Risk within levels of vulnerability		Risk within levels of vulnerability	
		*n* (%)	*n* (%)	Risk ratio (CIs)	*n* (%)	Risk ratio (CIs)	*n* (%)	Risk ratio (CIs)
Social isolation	Yes	57,218 (60)	17,370 (30)	1.15 (1.12, 1.17)	11,664 (20)	1.71 (1.66, 1.76)	7826 (14)	2.19 (2.1, 2.29)
	No	37,757 (40)	9980 (26)		4504 (12)		2356 (6)	
CALD	Yes	34,279 (36)	11,149 (33)	1.22 (1.19, 1.24)	6576 (19)	1.21 (1.18, 1.25)	4408 (13)	1.35 (1.3, 1.4)
	No	60,969 (64)	16,201 (27)		9592 (16)		5774 (10)	
Carer‐sustainability concerns	Yes	20,236 (21)	8095 (40)	1.55 (1.52, 1.59)	5206 (26)	1.75 (1.7, 1.81)	3308 (16)	1.78 (1.71, 1.85)
	No	74,739 (79)	19,255 (26)		10,962 (15)		6874 (9)	
War veterans	Yes	1032 (1)	339 (33)	1.14 (1.05, 1.25)	204 (20)	1.16 (1.03, 1.32)	136 (13)	1.23 (1.05, 1.44)
	No	93,943 (99)	27,011 (29)		15,964 (17)		10,046 (11)	
Aboriginal and Torres Strait Islanders	Yes	684 (1)	139 (20)	.7 (.61, .82)	151 (22)	1.3 (1.13, 1.5)	163 (24)	2.24 (1.96, 2.57)
	No	94,291 (99)	27,211 (29)		16,017 (17)		10,019 (11)	
Asylum seeker or refugee	Yes	398 (0)	160 (40)	1.4 (1.24, 1.58)	104 (26)	1.54 (1.3, 1.82)	97 (24)	2.29 (1.92, 2.72)
	No	94,557 (100)	27,190 (29)		16,064 (17)		10,085 (11)	
Gender diverse	Yes	127 (0)	31 (24)	.85 (.62, 1.15)	29 (23)	1.34 (.97, 1.85)	32 (25)	2.35 (1.74, 3.18)
	No	94,848 (100)	27,319 (29)		16,139 (17)		10,150 (11)	

Abbreviation: CIs, confidence intervals.

### 3.1 Sociodemographic characteristics

The study population was approved for one of the four HCP‐levels: Level‐1 (5%), Level‐2 (38%), Level‐3 (34%) and Level‐4 (24%). Average age was 82 (SD 7.6) years, and the majority were women (60%). Half the population was born in Australia; 22% were born in Southern and Eastern European countries. About a quarter of people (24%) had first languages other than English. Those living with family were more likely to be approved for HCP Level‐4. People residing in the lowest and the three highest IRSAD deciles were over‐represented in the study population—that is, they represented more than the expected 10% per decile. People in the highest decile (most advantaged/least disadvantaged) were more likely to be approved for HCP Level‐4 (36%), compared to other IRSAD deciles (<25%) (Table 2).

### 3.2 Care and support needs

As expected, the higher a person's care and support needs, the higher the HCP‐level for which they were approved (Table 3).

#### 3.2.1 Physical considerations

For activities‐of‐daily‐living, approximately half the population needed at least ‘some‐assistance’ (see Appendix S1 for definition) with walking (59%), showering (53%), medication management (57%), transfers (40%) and getting dressed (43%). People also commonly needed at least ‘some‐assistance’ for toileting (bladder (29%) and bowel (20%)) and eating (18%). People needing assistance for mobility and personal care tasks were more likely to be approved for a higher level HCP. A relatively small number of people needed ‘high‐level‐assistance’ (see Appendix S1 for definition); these individuals almost always required a HCP level‐3 or ‐4 (Table 3).

More than half of the population had poor vision and hearing, difficulty sleeping, had experienced falls in the last 12‐months and had recently experienced pain. Where a person was blind (%) or had issues with speech (8%), swallowing (14%), communication (37%) or food/fluid intake (41%), a higher level of HCP was approved (Table 3).

#### 3.2.2 Cognitive, behavioural and psychological considerations

Regarding cognitive concerns, 75% of people experienced some level of short‐term memory loss. When short‐term memory loss was recorded as ‘regular’ (23%) or ‘always’ (6%), HCP Level‐4 was approved. Disorientation to time, place or person was less frequent overall, but even if only ‘occasionally’ present (19%, 11% and 9%, respectively), HCP level‐3 or ‐4 was approved. Common behavioural concerns were impaired judgement (24%), agitation (21%), apathy (19%) and confusion (33%); even if present only ‘occasionally’ (18%, 18%, 14% and 24%, respectively), they were associated with HCP level‐3 or ‐4. Psychological concerns were frequent, with many experiencing depression (53%) and anxiety (58%) at least ‘occasionally’; however, these concerns influenced the approved HCP‐level to a lesser degree than cognitive and behavioural concerns (Table 3).

#### 3.2.3 Psychosocial considerations

Most people had a carer (81%), and 16% were themselves caring for someone else. Overall, 79% of people had at least one assessed vulnerability factor, and 44% reported at least one complexity indicator (Table 3). People who experienced any of the recorded vulnerability factors or complexity indicators were over‐represented among those approved for HCP level‐3 or ‐4.

#### 3.2.4 Health concerns and healthcare use

In all, 94% of all people assessed had three or more co‐morbidities (Table 3); however, the number of co‐morbidities did not appear to influence the level of HCP required (see Appendix S1 for definition of the number of co‐morbidities). Overall, 41% of the population had been hospitalised in the 3 months prior to assessment (Table 3).

### 3.3 Associations between vulnerability factors and complexity indicators

Vulnerability factors were positively associated with the three most prevalent complexity indicators (significant cognitive changes, self‐neglect and emotional/mental health issues), except for Aboriginal and Torres Strait Islander peoples (*n* = 684), who appeared less likely to have significant cognitive changes. People experiencing social isolation (*n* = 57,218) were more likely to report self‐neglect (RR 1.71, 95% CI 1.66–1.76) and emotional/mental health issues (RR 2.19, 95% CI 2.10–2.29). Where there were carer‐sustainability concerns (*n* = 20,236), the care recipient was more likely to have significant cognitive changes (RR 1.55, 95% CI 1.52–1.59), self‐neglect (RR 1.75, 95% CI 1.70–1.81) or an emotional/mental health issue (RR 1.78, 95% CI 1.71–1.85).

## 4 DISCUSSION

This observational study had four main findings. First, similar to other Australian studies about similar population groups,^14^, ^16^, ^17^ the population was socio‐demographically diverse across age, culture, living‐situation and socio‐economics. Second, the study population had advanced care and support needs, including personal care, cognitive and behavioural changes, and physical concerns such as with food/fluid intake and falls. While there is some understanding amongst clinicians, aged‐care providers and policymakers of the types of needs experienced by people requiring home‐based aged‐care—particularly in terms of commonly used service types^6^, ^7^—this study provides new evidence and population‐level information about the comprehensive range, and importantly the prevalence, of underlying needs experienced by Victorians, who make up a quarter of the Australian population requiring home‐based aged‐care.^17^ Third, the population had a range of vulnerabilities and complexities, with many having more than one vulnerability factor or complexity indicator, which tended to be associated, further increasing complexity—such insights have not been reported before in other Australian studies. Fourth, the expected high level of co‐morbidity amongst the population was very evident.^14^

While the study's sociodemographic findings mirrored those of other studies, a new and concerning sociodemographic issue identified in this study was inequity in HCP approval rates between socio‐economic groups. It was not possible to directly explore reasons for this finding, especially since the study population only included people approved for a HCP, and not those who applied but were deemed ineligible. However, lower health literacy amongst people from lower socio‐economic groups is a potential explanation.^27^ In the context of home‐based aged‐care, this could translate to people of lower socio‐economic backgrounds not knowing about such support and, therefore, seeking a HCP in lower numbers than those from more advantaged areas. It could also reflect the stronger advocacy people from higher socio‐economic backgrounds can bring to discussions with their aged‐care assessor due to their higher health literacy, which could result in a higher level of HCP being approved.

The Royal Commission into Aged Care Quality and Safety found that accessing the aged‐care system can be ‘…time‐consuming, overwhelming, frightening and intimidating’.^28^ ^(p.65)^ This finding highlights why the home‐based aged‐care system, both the current programs and the planned reformed home‐based aged‐care program, must be designed to be easy to navigate and access for older people from a range of different sociodemographic backgrounds so that all older people equitably receive the benefits of home‐based aged‐care. Of note, the ‘care finder’ role has been established as an ongoing part of Australia's aged‐care system to assist people with health literacy challenges in navigating the aged‐care system.^29^

More than half (57%) of the study population (those approved for Level‐3 and ‐4 HCPs) commonly required support for advanced personal care and clinical needs, which has implications for the home‐based aged‐care system. This new finding regarding the high prevalence of such needs, compares favourably to Canadian and US studies, in which high rates of personal care and clinical needs necessitating nursing and primary care support were found in people requiring home‐based aged‐care.^19^, ^20^, ^21^ The high prevalence of personal care and clinical needs in this Victoria study population indicates that the proposed home‐based aged‐care program reform should be configured to provide timely and adequate personal care and clinical services to manage such high care and support needs. The required workforce skills and skill mix to competently identify and address such needs for the duration a person receives home‐based aged‐care must also be carefully considered. This might include service‐delivery models that incorporate clinical leadership, clinical support roles and multidisciplinary team‐based care structures.^30^ Indeed, this finding supports recommendations in the revised Aged Care Quality Standards,^31^ especially the Clinical Care Standard, stipulating such assessment, care planning and workforce considerations.

Aside from specific care and support needs, the overall ‘complexity’ of the study population was very apparent, with many individuals experiencing more than one vulnerability and/or complexity factor, which tended to be associated, further increasing overall complexity. The literature suggests numerous factors contribute to complexity, including medical, psychological, social and lifestyle factors, which accumulate over time.^32^ The presence of complex needs amongst people requiring home‐based aged‐care drives an imperative to structure care delivery models to support such complexity, which can make the reality of home‐based aged‐care provision discernibly messy.^33^ Complexity must, therefore, be considered when reforming the home‐based aged‐care program for older Australians. This might entail ensuring funding is available for sufficient care management support for people experiencing high‐intensity complexity, to gradually work through and address their multiple care and support needs. For certain subgroups of older people, for example those who are financially or socially disadvantaged or have poor mental health,^34^ care management monitoring and support can be just as important as direct services.^35^ Workforce considerations are again also crucial here. Supporting individuals with complex care and support needs requires care managers to have a strong understanding of the issues of ageing, experience in aged‐care assessment and care‐planning, trust and rapport building, and the ability to work within multidisciplinary care teams that, importantly, engage the older person themselves.^36^

The high prevalence of co‐morbidity and hospital use across the study population is also notable, and aligns with Australian research by Inacio et al.,^14^ who found declining health status amongst a similar population from 2006 to 2015. International studies also show high levels of morbidity amongst similar population groups.^19^, ^20^, ^21^ Managing multiple co‐morbidities in older people can present considerable challenges, often leading to hospitalisation, placing significant demand on the hospital system.^37^ Good health and care outcomes for this population are more likely to be achieved through multi‐disciplinary, team‐based, person‐centred management in the primary health‐care setting, rather than hospitals.^38^, ^39^ Therefore, creative approaches to better integrate the proposed new home‐based aged‐care system with the broader health‐care system are essential.

This study's key strength is that it has been conducted using a population‐based dataset (almost 100,000 people) comprising all Victorians approved for a HCP, providing a unique opportunity to describe the population's characteristics and needs comprehensively. Because the entire Victorian population of interest was included, no sampling bias is anticipated. While we acknowledge the study findings cannot be extrapolated to all Australian states, they are still likely to be informative for states with similar sociodemographics such as New South Wales and South Australia, particularly considering that the full Australian population is assessed in a consistent way under the Aged Care Assessment Program.^22^

The study shares limitations with other research using routinely collected datasets. First, data‐entry inconsistencies for a small number of variables necessitated some assumptions in data interpretation. Poor vision, poor hearing, speech issues and decision‐making support requirements started with ‘yes/no’ questions, with follow‐up questions collecting more detailed information for those reporting ‘yes’. Because the skip‐logic did not work properly for the follow‐up questions, we decided to report responses for the most severe condition only (i.e. blind, deaf, assistance required for health/lifestyle decisions and financial decisions). These decisions could have resulted in under‐ or over‐representation of some care and support needs. Second, possible information bias during data collection may have contributed to under‐reporting of care and support needs (e.g. an individual might under‐report their care issues to their assessor to avoid aged‐care) or over‐reporting (e.g. assessors with certain clinical experiences might be more sensitive to certain needs). Lastly, while this was a population‐level study of people nominating for eligibility assessment for government‐funded aged‐care services, self‐selection bias cannot be ruled out, as people from vulnerable population groups might not readily nominate for assessment and, therefore, be under‐represented in the study population.

## 5 CONCLUSIONS

This study has reported the diverse sociodemographic characteristics of the home‐based aged‐care population. It has also provided new and comprehensive insights into the range and prevalence of day‐to‐day mobility, functional, physical, cognitive, behavioural, psychological and psychosocial care and support needs experienced by older Victorians requiring home‐based aged‐care, showing that the study population commonly had advanced and complex needs. Furthermore, it has provided new evidence about inequitable HCP approval rates between different socio‐economic sub‐groups. These findings could inform: (1) home‐based aged‐care service‐delivery models to ensure pathways exist to meet the range and prevalence of care and support needs experienced by the population; and (2) clinical and non‐clinical workforce skill‐mix requirements to efficiently address the population's care and support needs.

## FUNDING INFORMATION

The first author was supported by a Deakin University Postgraduate Research Scholarship to undertake this research. The Australian College of Nursing provided additional support via the Centaur Nurses Memorial Scholarship 2022, as did the Australian Nursing and Midwifery Federation (Victorian Branch) through the Annual Higher Education and Research Grant 2023.

## CONFLICT OF INTEREST STATEMENT

We declare the following: Martin Hensher is a board director of Glenview Community Services, a Tasmanian not‐for‐profit residential and home‐based aged care provider; Rachel McKittrick is an Assessment Clinician with the Kingston Aged Care Assessment Service (Monash Health) in Victoria.

## Supporting information


Appendix S1

Appendix S2

## Data Availability

The National Screening and Assessment Form (NSAF) dataset (2022 version) used for this study was supplied by the Australian Institute of Health and Welfare under a confidentiality agreement and so cannot be made freely available. The data is stored in the National Aged Care Data Clearinghouse under the custodianship of the AIHW. Access requests to these data should be made to AIHW via their ‘data on request’ processes (https://www.aihw.gov.au/about‐our‐data/accessing‐data‐through‐the‐aihw/data‐on‐request).
