# Supplementary material for: Characteristics, care and support needs of older Victorians requiring a government‐funded Home Care Package: An observational study
Source: Australas J Ageing. 2025 Jan 12;44(1):e13400. doi: 10.1111/ajag.13400 (PMC11903932; doi:10.1111/ajag.13400)
Supplement: Supplementary file 1 — Appendix S1 [file AJAG-44-0-s002.docx]

**Supplementary File 1: Variable definitions as contained in the National Screening and Assessment Form (NSAF) and required to be considered by Assessors when assessing a person’s eligibility for a Home Care Package^1^**

| **Variable** | **Variable definition** | **Response options  (where the response options are ‘yes’ or ‘no’, it is the ‘yes’ response that is relevant for analysis, unless otherwise indicated)** |
| --- | --- | --- |
| **Approval types/outcome variable:** | | |
| Home Care Package | Home Care Package (HCP) approval is the outcome of an aged care assessment, where an individual meets specific eligibility criteria, as set out by the Aged Care Act 1997. The HCP program consists of four HCP levels, which fund increasing levels of flexible and personalised direct care services that an older person requires to remain living safely at home. Level 1 is the lowest level of HCP for people for people with basic level care and support needs. Level 4 is the highest level of HCP for people with high level care and support needs. | 1, 2, 3 or 4 |
| **Socio-demographic characteristics:** | | |
| Age (years) | Age was calculated based on the individual's month and year of birth, as provided in the NSAF dataset. | - |
| Gender | Individual's reported gender was provided in the dataset | Male or female only |
| Birth country (broad groupings) | Individual level birth countries were provided in the dataset, which were assigned to the relevant major group of Australian Bureau of Statistics (ABS) Standard Australian Classification of Countries (2016)^2~~!~~^, with some slight variation to this listing | Australia, Other Oceania and Antarctica, UK and Ireland, Other North-West Europe, Southern and Eastern Europe, North Africa and the Middle East, Sub-Saharan Africa, South-East Asia, North-East Asia, Southern and Central Asia, Americas |
| Preferred language | Individual level preferred language was provided in the dataset, which were assigned to the relevant major group of Australian Bureau of Statistics (ABS) Australian Standard Classification of Languages (2016).^2^ Languages spoken by >1% of the dataset population have been listed, with those spoken by 1% or less of the database population grouped into ‘Other languages’ | - |
| Marital status | Individual level marital status | Married (registered/de facto), widowed, divorced/separated, never married |
| Living situation | Individual level living situation | With partner, lives alone, with family, with others, not applicable |
| Accommodation type | Individual level type of accommodation | Client owns, family/relation owns, private rental, independent living, public housing, institutional (including residential aged care and supported residential services), homeless or temporary/transition housing, other community setting (including Indigenous communities) |
| Aged Care Planning Region (ACPR) | Aged care services in Australia tend to be funded and delivered in regions called ACPRs - there are nine within Victoria across metropolitan and regional areas | *Melbourne metropolitan areas:* southern metropolitan, eastern metropolitan, northern metropolitan, western metropolitan *Victorian regional areas:* Barwon-South Western, Gippsland, Loddon-Mallee, Grampians, Hume |
| Geographical remoteness within Victoria - Modified Monash Model (MMM) location | A Commonwealth Department of Health and Aged Care Australian Statistical Geography Standard classification of geographical remoteness of towns/suburbs, also taking into account population size^3~~#~~^ | Metro, regional, large rural, medium rural, small rural, remote, very remote |
| Index of Relative Socio-economic Advantage and Disadvantage (IRSAD) deciles | Postcodes are rated by the Australian Bureau of Statistics according to the level of relative advantage and disadvantage that people who live in that postcode are likely to experience. The IRSAD is a measure summarising information about the economic and social conditions of people and households in particular postcodes, including both relative advantage and disadvantage measures. A low score means there is a relatively more disadvantage than advantage. A high score means there is relatively more advantage than disadvantage^4^ | The rating is presented in Victorian deciles, where the first decile is the lowest and the 10th decile is the highest: 1st (0-10%), 2nd (11-20%), 3rd (21-30%), 4th (31-40%), 5th (41-50%), 6th (51-60%), 7th (61-70%), 8th (71-80%), 9th (81-90%), 10th (91-100%) |
| **Care and support needs:** | | |
| **Physical considerations: activities of daily living (NB: assistance can be required for either physical or cognitive reasons)** | | |
| ***Mobility:*** |  |  |
| Walking | Whether the individual can walk, assessing across indoor, outdoor and community mobility - considering falls risk, shortness of breath, foot problems etc | No assistance Some assistance (help of one person or a walking frame is required) High level of assistance (wheelchair bound and can't self-propel, bed bound and/or needs help of more than one person to mobilise) |
| Transfers | Whether the individual can move from place to place. Includes difficulties with all types of transfers - considering use of the shower, toilet, bed, chairs | No assistance Some assistance (ranging from use of an aid/equipment, verbal prompting, to the help of one or two people) High level of assistance (has no sitting balance and needs full assistance from others or the use of a hoist etc to transfer) |
| ***Personal care:*** |  |  |
| Showering/bathing | Whether the individual can shower, bath or bathe themselves - considering hair care, feet/nail care, managing taps/water temperature, teeth/oral care, confidence to manage balance while showering etc | No assistance Some assistance (either hands on or standby assistance to manage any element of the showering/bathing process) High level of assistance (needs total assistance with preparing and wishing/drying or uses bed sponges only) |
| Dressing | Whether the individual can dress themselves - considering appropriate clothing choice, physical assistance requirement to dress/undress for any item of clothing or footwear | No assistance Some assistance (needs some hands on or standby assistance to manage any element dressing) High level of assistance (completely unable to dress themselves) |
| Toileting (bladder) | This refers to the personal care aspect of toileting & management of any incontinence issues (bladder) | No assistance Some assistance (such as to move on/off the toilet, manage continence aids/catheter etc, clothing adjustment or personal hygiene after use of bladder) High level of assistance (completely unable to manage toileting without full assistance) |
| Toileting (bowel) | This refers to the personal care aspect of toileting & management of any incontinence issues (bowel) | No assistance Some assistance (such as to move on/off the toilet, manage continence aids/catheter etc, clothing adjustment or personal hygiene after use of bowel) High level of assistance (completely unable to manage toileting without full assistance) |
| Medication management | Whether the individual can take their own medications | No assistance (self-medicates right doses at the right time) Some assistance (can take medications if prepared for them by someone else including if they are in a dosette box or blister pack or if they are reminded to take them) High level of assistance (incapable of organising any aspect of medication management or tends to be non-compliant with medication regime) |
| Eating/feeding | Whether the individuals can feed themselves - considering meal setup, cutting up of food which could be related to hand dexterity, adaptive cutlery requirements, prompting being needed to encourage eating etc | No assistance (once food is in reach) Some assistance (needs some hands on or standby assistance to manage any element eating) High level of assistance (completely unable to feed themselves, including individuals fed via nasogastric or Percutaneous Endoscopic Gastrostomy) |
| ***Domestic tasks:*** |  |  |
| Meal preparation | Whether the individual can prepare their own meals - considering safety to use appliances in the kitchen, plan meals to eat, dexterity to cut up meat or vegetables | No assistance  Some assistance (can prepare some light meals but cannot cook a full meal themselves, but can re-heat pre-prepared food) High level of assistance (completely unable to participate in any aspect of meal preparation) |
| Housework | Whether the individual can do housework - cleaning, vacuuming, change bed linen, other general housekeeping | No assistance  Some assistance (can do light housework like dusting, dishes, laundry but needs help with heavier tasks like vacuuming, hanging clothes on the line, changing bed linen) High level of assistance (completely unable to participate in any housework) |
| ***Community access:*** |  |  |
| Transport | Whether the individual can get to places out of walking distance - considering physical assistance or supervision requirements | No assistance  Some assistance (needs help from another person to arrange transport, to get in/out of the car/bus etc, and may need help to navigate where they are travelling to) High level of assistance (needs help from at least one other person, and can only travel in an emergency and/or in specialised vehicles) |
| Shopping | Whether the individual can go shopping for groceries or clothes, once they have transportation organised - considering mobility, vision etc | No assistance  Some assistance (needs help from another person with any aspect of shopping including making payment, reading labels, carrying items) High level of assistance (completely unable to participate in any shopping activities) |
| Handling money/paying bills | Whether the individual can handle money - considering bill payment, paying for groceries etc | No assistance  Some assistance (can manage day to day purchases but needs help with banking, bills or other major purchases) High level of assistance (incapable of handling money or finances) |
| Home maintenance concerns | Whether the individual can keep their home in a safe and habitable conditions, so that the home is safe to mobilise/function within (including in the garden) | Yes or no *(no response for this variable is relevant for analysis)* |
| **Physical considerations: other care concerns** | |  |
| Sensory concerns: |  |  |
| Poor vision | Low vision or blindness, one or both eyes | Yes or no |
| Blindness |  | In the NSAF, assessors can select either blindness, low vision or not applicable. There were issues with this variable - blindness, low vision and not applicable together, do not equal those who have ‘poor vision’ as per the above variable. The decision was made to only tabulate blindness, the most significant impairment. |
| Poor hearing | Poor hearing or deafness, one or both ears | Yes or no |
| Deafness |  | In the NSAF, assessors can select either poor hearing or deafness or not applicable. There were issues with this variable - poor hearing, deafness and not applicable together, do not equal those who have ‘poor hearing’ as per the above variable. The decision was made to only tabulate deafness, the most significant impairment. |
| Speech issues | Any difficulty with speaking or swallowing - particularly relevant for individuals with Parkinson's Disease, Alzheimer's Disease, stroke, certain concerns, motor neurone disease | Yes or no |
| Swallowing problems | Whether the individual has any difficulties swallowing (dysphagia) - food, fluids, saliva | Yes or no |
| Communication difficulties | The individual perceives difficulty with communicating - includes over the phone as well as face to face |  |
| Oral health concerns | Whether the individual has any concerns with their teeth, mouth or dentures that needs addressing |  |
| Food, fluid and/or weight concerns | Whether the individual has any issues with their appetite (decreased appetite/difficulty with digesting food); fluid intake (adequate daily fluid intake or on a fluid restriction); weight (especially unintentional weight loss) |  |
| Major skin conditions | Whether the individual has major skin concerns - pressure injury, other skin ulceration, other skin tears/cuts/lesions/ bruising/rashes/itching/eczema |  |
| Has difficulty sleeping | Whether the individual experiences difficulty sleeping |  |
| Falls/slips/trips in last 12 month | Whether the individual has had any falls in the past 12 months |  |
| Has inadequate physical activity | Whether the individual regularly undertakes 30 minutes of physical activity | Yes or no *(no response for this variable is relevant for analysis)* |
| Experienced bodily pain in last 4 weeks | Whether the individual has experienced pain/discomfort in the past 4 weeks | Yes or no |
| **Cognitive/behavioural/psychological considerations:** | | |
| **Decision making assistance:** | | |
| Health/lifestyle decisions | Whether the individual needs someone to assist them to make health or lifestyle decisions | In the NSAF, assessors can select neither or both health/lifestyle decisions and financial decisions. |
| Financial decisions | Whether the individual needs someone to assist them to make financial decisions |  |
| **Specific cognitive care concerns:** | | |
| Short term memory loss | Experiences short term memory loss | Never (no evidence of this concern) Occasionally (e.g. weekly) Regularly (e.g. one or more times a week) Always (daily) Unable to determine (if the person or their carer/others cannot communicate any information about this concern) |
| Long term memory loss | Experiences long term memory loss |  |
| Disorientation - time | Unable to identify the time, day, date or year |  |
| Disorientation - place | Unable to identify their address or where they are currently located |  |
| Disorientation - person | Unable to identify persons such as family/friends |  |
| Hallucinations | Experiences auditory, visual, olfactory, gustatory, tactile hallucinations; experiencing false or erroneous beliefs that usually involve a misinterpretation of perceptions or experiences |  |
| **Specific behavioural care concerns:** | | |
| Impaired judgement | Unable to make good decisions due to underlying medical issue(s) | Never (no evidence of this concern) Occasionally (e.g. weekly) Regularly (e.g. one or more times a week) Always (daily) Unable to determine (if the person or their carer/others cannot communicate any information about this concern) |
| Risky behaviour | Displays behaviours that puts themselves or others at risk e.g. in use of kitchen or other household appliances |  |
| Verbal aggression | May yell, scream and/or threaten |  |
| Physical aggression | May hit, scratch, bite, push, shove, throw things, or use weapons |  |
| Agitation | Can become extremely emotionally disturbed |  |
| Apathy | Experiences an absence or suppression of passion, emotion or excitement |  |
| Wandering | Moves about without a definite destination or purpose – inside or outside the home |  |
| Resistive behaviour | Can resist/oppose help or caregiving tasks such as taking medication and eating |  |
| Confusion | Has altered consciousness, attention, cognition and perception, that may have come on quickly, or slowly over time and be an ongoing state, depending on the cause |  |
| **Specific psychological care concerns:** | | |
| Insomnia | Has persistent difficulty initiating or maintaining sleep | Never (no evidence of this concern) Occasionally (e.g. weekly) Regularly (e.g. one or more times a week) Always (daily) Unable to determine (if the person or their carer/others cannot communicate any information about this concern) |
| Depression | Experiences symptoms such as long periods of feeling lonely, overwhelming feelings of being unable to keep going or regular tears |  |
| Anxiety | Experiences an unpleasant state of inner turmoil, often accompanied by nervous behaviour such as pacing back & forth, somatic complaints and rumination |  |
| Loneliness | Expresses feeling lonesome, alone, deserted or isolated from friends/family/community |  |
| Social isolation | The individual lacks engagement with others, has a minimal number of social contacts and is deficient in fulfilling quality relationships |  |
| **Psychosocial considerations:** | | |
| **Social support situation/carer status:** | | |
| Has a carer | Is receiving support from a carer, family member, friend and/or neighbour that is not associated with a service provider or paid service | Yes or no |
| Is themselves a carer | Is supporting another person, such as with their activities of daily living and/or self-care |  |
| **Vulnerability factors (which might indicate a person needs additional support to access ‘mainstream’ aged care services, and recorded based on the Assessor's clinical judgement)** | | |
| Socially isolated | Lacks engagement with others, has a minimal number of social contacts and is deficient in fulfilling quality relationships | Yes or no |
| Culturally and linguistically diverse (CALD) | Culturally and linguistically or ethnically diverse individual |  |
| Carer sustainability concerns | There is risk of a change in family/carer support arrangements, possibly due to high levels of carer stress. If the change in family/carer arrangements were to occur, the safety and wellbeing of the individual needing care at home, would be compromised, potentially resulting in residential care being required |  |
| War veterans | War veteran |  |
| Aboriginal and Torres Strait Islander | Identifies as being Aboriginal and/or Torres Strait Islander |  |
| Asylum seeker or refugee | Asylum seeker/refugee/recent migrant without support to navigate support systems where these are required |  |
| Gender diversity | Lesbian, gay, bisexual, transgender, intersex or other gender diverse individuals |  |
| Multi-vulnerability | This is a derived variable – number of vulnerability factors identified for each person | 0, 1, 2, 3+ |
| **Complexity indicators:** | | |
| Significant cognitive changes | The individual has declining cognitive health/memory issues/confusion that significantly limits self-care capacity, requires intensive/constant or near constant supervision, prompting and/or standby and/or actual physical assistance with self-care or other activities, and may not be able to be left alone for longer than 5 minutes | Yes or no |
| Self-neglect | The individual is or is at risk of self-neglecting personal care and/or safety, which may cause them or others harm |  |
| Emotional or mental health issues | The individual has emotional or mental health issues that affect their ability to cope with daily living and/or stressful life events, and place them at risk of being marginalised by mainstream services, noting that some mental health concerns can be cyclic and if not managed can lead to periods of rapid deterioration |  |
| Financial disadvantage | The individual experiences financial issues, which may be a result of high medical or accommodation expenses, living beyond their means that limits capacity to pay for essential home-based services, or behaviours such as gambling, which then threatens their ability to remain safely at home |  |
| Inadequate housing | The individual is living in inadequate housing, housing that does not suit their needs, has precarious tenure or housing rights, is living in unstable housing such a boarding house or staying with friends/relatives, or is already homeless, and this situation impacts on their wellbeing and ability to remain living in the community |  |
| Risk of, or suspected or confirmed abuse | There is a risk of, or suspected, or confirmed abuse that puts the person at risk of harm and/or neglect, noting this may be intentional or unintentional and may be caused by another person with whom the person has a relationship of trust |  |
| Drug and alcohol use likely to cause harm to self or others | The individual is likely to cause harm to themselves or others and impede their access to aged care services due to misuse of drugs (including prescription drugs) and alcohol |  |
| History of institutionalisation | The individual has experienced adverse effects of institutionalisation and/or systems abuse, and are refusing assistance or services when these are clearly needed to maintain safety and wellbeing – includes those who have been in prisons, foster care, residential care, out of home care, Forgotten Australians, Stolen Generations |  |
| Multi-complexity | This is a derived variable – number of complexity indicators identified for each person | 0, 1, 2, 3+ |
| **Safety concerns:** | | |
| Home safety concerns | There are identified risks, hazards or concerns for the individual due to their home set up – may include access issues (broken steps, uneven footpath or overgrown lawns/garden), pets or signs of clutter/hoarding, difficulties accessing items such as cupboards, clothesline, letterbox or driveway, home maintenance issues | Yes or no |
| Personal safety concerns | The individual has issues maintaining their own personal safety, due to issues such as family violence (including physical danger or other threats), abuse (including physical, emotional, financial), presence of weapons, or other reported issues |  |
| **Health concerns and healthcare use:** |  |  |
| Health concerns | Whether the person has any health conditions, or other signs & symptoms, which impact on the person’s need for support with activities of daily living and social participation | Specific health conditions were listed in the NSAF dataset; however, these were merged into groupings of similar type conditions for the purpose of this analysis. Selected re-grouped health concerns were listed in Table 3. The full list of re-grouped conditions is provided in this Supplementary File in the below table. |
| Muti-morbidity | This is a derived variable – number of health conditions identified for each person | 0, 1-2, 3-4, 5-6, 7-8, 9+ |
| **Healthcare use:** | | |
| Sees a GP regularly | Whether the person sees a GP regularly | Yes or no |
| Using other clinical services | Whether the person accesses any short term or ongoing clinical services, which might include palliative care, mental health services, diabetes education, medical specialists, and hospital outpatient services |  |
| Health literacy support required | The person has the ability to read, understand and use healthcare information to make informed decisions about health and have the ability to follow treatment instructions where required |  |
| Hospitalisations | Whether the person has been admitted to hospital in the past 3 months, or is currently in hospital (at the time of assessment) | Yes, has been admitted to hospital in the past 3 months or is currently in hospital (at time of assessment) |

1. My Aged Care. *National Screening and Assessment Form (NSAF) User Guide: a guide to the information required to be considered and recorded during the My Aged Care assessment process*. 2018. <https://www.health.gov.au/sites/default/files/documents/2020/01/my-aged-care-national-screening-and-assessment-form-user-guide_0.pdf>

2. Australian Bureau of Statistics. Classifications: 1-18 of 18 classifications. Updated 22 November 2022. Accessed 15 August, 2024. <https://www.abs.gov.au/statistics/classifications>

3. Department of Health and Aged Care (DoHAC). Modified Monash Model. Updated 12 December 2023. Accessed 15 August, 2024. <https://www.health.gov.au/topics/rural-health-workforce/classifications/mmm#about-the-modified-monash-model>

4. Australian Bureau of Statistics. 2033.0.55.001 - Census of Population and Housing: Socio-Economic Indexes for Areas (SEIFA), Australia, 2016. Updated 21 March 2023. Accessed 12 September, 2024. <https://www.abs.gov.au/ausstats/abs@.nsf/Lookup/by%20Subject/2033.0.55.001~2016~Main%20Features~IRSAD~20>
