# Supplementary material for: Characteristics, care and support needs of older Victorians requiring a government‐funded Home Care Package: An observational study
Source: Australas J Ageing. 2025 Jan 12;44(1):e13400. doi: 10.1111/ajag.13400 (PMC11903932; doi:10.1111/ajag.13400)
Supplement: Supplementary file 2 — Appendix S2 [file AJAG-44-0-s001.docx]

**Supplementary file 2:** Prevalence of all socio-demographic characteristics, care and support needs, health concerns and healthcare use, as available in the National Screening and Assessment Form (NSAF) study dataset, for people approved for a Home Care Package (HCP) between January 2019 and June 2022: for all and by HCP level

|  | **All** | **HCP L1** | **HCP L2** | **HCP L3** | **HCP L4** |
| --- | --- | --- | --- | --- | --- |
| **Approved HCP level:** | **n (%)** | **n (%)** | **n (%)** | **n (%)** | **n (%)** |
| **HCP approval rates:** | 94,975 (100) | 4,883 (5) | 35,723 (38) | 32,031 (34) | 22,338 (24) |
| **Sociodemographic characteristics:** |  |  |  |  |  |
| **Age (years)** |  |  |  |  |  |
| Mean (SD) | 82 (7.6) | 80 (6.9) | 82 (7.3) | 82 (7.8) | 83 (8.1) |
| Min - max | 45 - 109 | 51 - 102 | 50 - 106 | 47 - 105 | 45 - 109 |
| <65yo (not the target population of the assessment or this study ^*^) | 496 (0.5) | 18 (3.6) | 155 (31.3) | 169 (34.1) | 154 (31.1) |
| **Gender ^a^** |  |  |  |  |  |
| Female | 57,253 (60) | 2,810 (5) | 22,231 (39) | 19,450 (34) | 12,762 (22) |
| Male | 37,719 (40) | 2,072 (6) | 13,491 (36) | 12,581 (33) | 9,575 (25) |
| **Birth country (broad groupings) ^b^** |  |  |  |  |  |
| Australia | 49,115 (52) | 2,650 (5) | 18,851 (38) | 16,735 (34) | 10,879 (22) |
| Southern & Eastern Europe | 20,459 (22) | 787 (4) | 7,338 (36) | 6,888 (34) | 5,446 (27) |
| United Kingdom & Ireland | 7,637 (8) | 441 (6) | 2,915 (38) | 2,574 (34) | 1,707 (22) |
| Other North West Europe | 3,159 (3) | 177 (6) | 1,155 (37) | 1,081 (34) | 746 (24) |
| North Africa & the Middle East | 2,978 (3) | 123 (4) | 1,063 (36) | 1,005 (34) | 787 (26) |
| North-East Asia | 2,953 (3) | 307 (10) | 1,304 (44) | 825 (28) | 517 (18) |
| South-East Asia | 2,480 (3) | 112 (5) | 848 (34) | 861 (35) | 659 (27) |
| South & Central Asia | 2,206 (2) | 109 (5) | 763 (35) | 734 (33) | 600 (27) |
| Other Oceania & Antarctica | 954 (1) | 39 (4) | 336 (35) | 328 (34) | 251 (26) |
| Sub-Saharan Africa | 878 (1) | 47 (5) | 275 (31) | 298 (34) | 258 (29) |
| Americas | 681 (1) | 19 (3) | 241 (35) | 248 (36) | 173 (25) |
| **Preferred language ^c^** |  |  |  |  |  |
| English | 72,297 (76) | 3,950 (6) | 27,624 (38) | 24,505 (34) | 16,218 (22) |
| Greek | 5,037 (5) | 229 (5) | 1,787 (36) | 1,639 (33) | 1,382 (27) |
| Italian | 4,867 (5) | 136 (3) | 1,712 (35) | 1,585 (33) | 1,434 (30) |
| Chinese | 3,209 (3) | 318 (10) | 1,380 (43) | 916 (29) | 595 (19) |
| South Slavic | 1,631 (2) | 30 (2) | 524 (32) | 591 (36) | 486 (30) |
| Middle Eastern Semitic | 1,262 (1) | 34 (3) | 466 (37) | 408 (32) | 354 (28) |
| Mon-Khmer | 1,019 (1) | 32 (3) | 333 (33) | 366 (36) | 288 (28) |
| Other languages | 3,701 (4) | 73 (2) | 1,131 (31) | 1,370 (37) | 1,127 (31) |
| **Marital status ^d^** |  |  |  |  |  |
| Married (registered/de facto) | 45,849 (48) | 3,083 (7) | 17,353 (38) | 14,701 (32) | 10,712 (23) |
| Widowed | 32,851 (35) | 1,048 (3) | 12,021 (37) | 11,666 (36) | 8,116 (25) |
| Divorced/separated | 10,465 (11) | 469 (5) | 4,068 (39) | 3,703 (35) | 2,225 (21) |
| Never married | 3,952 (4) | 189 (5) | 1,491 (38) | 1,389 (35) | 883 (22) |
| **Living-situation ^e^** |  |  |  |  |  |
| With partner | 39,675 (42) | 2,787 (7) | 15,379 (39) | 12,620 (32) | 8,889 (22) |
| Lives alone | 37,342 (39) | 1,481 (4) | 15,115 (41) | 13,051 (35) | 7,695 (21) |
| With family | 15,305 (16) | 495 (3) | 4,262 (28) | 5,420 (35) | 5,128 (34) |
| With others | 1,984 (2) | 86 (4) | 729 (37) | 693 (35) | 476 (24) |
| Not applicable | 413 (0) | 19 (5) | 118 (29) | 160 (39) | 116 (28) |
| **Accommodation type ^f^** |  |  |  |  |  |
| Client Owns | 65,865 (69) | 3,476 (5) | 24,888 (38) | 21,891 (33) | 15,610 (24) |
| Family/Relation Owns | 8,666 (9) | 351 (4) | 2,842 (33) | 3,004 (35) | 2,469 (29) |
| Private Rental | 7,913 (8) | 377 (5) | 3,047 (39) | 2,760 (35) | 1,729 (22) |
| Independent Living | 6,185 (7) | 407 (7) | 2,624 (42) | 2,059 (33) | 1,095 (18) |
| Public Comm Housing | 4,704 (5) | 192 (4) | 1,698 (36) | 1,755 (37) | 1,059 (23) |
| Institutional inc Residential Aged care & Supported Residential Service | 760 (1) | 29 (4) | 253 (33) | 284 (37) | 194 (26) |
| Homeless or temporary/transition housing | 715 (1) | 34 (5) | 300 (42) | 227 (32) | 154 (22) |
| Other community setting inc Indigenous community | 76 (0) | 10 (13) | 29 (38) | 21 (28) | 16 (21) |
| **Aged care planning region (ACPR) ^g^** |  |  |  |  |  |
| *Melbourne metropolitan areas:* |  |  |  |  |  |
| Southern Metro | 21,532 (23) | 443 (2) | 6,995 (33) | 8,496 (40) | 5,598 (26) |
| Eastern Metro | 20,209 (21) | 2,339 (12) | 8,411 (42) | 5,441 (27) | 4,018 (20) |
| Northern Metro | 14,559 (15) | 556 (4) | 5,710 (39) | 4,649 (32) | 3,644 (25) |
| Western Metro | 10,446 (11) | 113 (1) | 3,643 (35) | 3,759 (36) | 2,931 (28) |
| *Victorian Regional areas:* |  |  |  |  |  |
| Barwon-South Western | 7,877 (8) | 166 (2) | 2,929 (37) | 2,832 (36) | 1,950 (25) |
| Gippsland | 7,513 (8) | 524 (7) | 2,540 (34) | 2,606 (35) | 1,843 (25) |
| Loddon-Mallee | 4,833 (5) | 250 (5) | 1,888 (39) | 1,548 (32) | 1,147 (24) |
| Grampians | 4,422 (5) | 118 (3) | 1,858 (42) | 1,660 (38) | 786 (18) |
| Hume | 3,055 (3) | 355 (12) | 1,581 (52) | 839 (28) | 280 (9) |
| **Geographical remoteness (MMM code) within Victoria ^h^** |  |  |  |  |  |
| Metro | 69,671 (73) | 3,470 (5) | 25,701 (37) | 23,469 (33) | 17,031 (24) |
| Regional | 6,364 (7) | 250 (4) | 2,486 (39) | 2,249 (35) | 1,379 (22) |
| Large rural | 5,984 (6) | 387 (7) | 2,555 (43) | 1,928 (32) | 1,114 (19) |
| Medium rural | 6,395 (7) | 416 (7) | 2,521 (39) | 2,146 (34) | 1,312 (21) |
| Small rural, remote & very remote | 6,516 (7) | 360 (6) | 2,443 (38) | 2,225 (34) | 1,488 (23) |
| **IRSAD (Victorian deciles) ^1^ ^i^** |  |  |  |  |  |
| 1st (least advantaged/most disadvantage) | 14,041 (15) | 598 (4) | 5,320 (38) | 5,022 (36) | 3,101 (22) |
| 2nd | 6,758 (7) | 324 (5) | 2,771 (41) | 2,345 (35) | 1,318 (20) |
| 3rd | 8,163 (9) | 401 (5) | 3,132 (38) | 2,769 (34) | 1,861 (23) |
| 4th | 6,021 (6) | 226 (4) | 2,148 (36) | 2,162 (36) | 1,485 (25) |
| 5th | 9,126 (10) | 370 (4) | 3,376 (37) | 3,317 (36) | 2,063 (23) |
| 6th | 5,218 (6) | 228 (4) | 2,008 (39) | 1,787 (34) | 1,195 (23) |
| 7th | 8,595 (9) | 448 (5) | 3,344 (39) | 2,828 (33) | 1,975 (23) |
| 8th | 12,231 (13) | 944 (8) | 4,879 (40) | 3,811 (31) | 2,597 (21) |
| 9th | 14,071 (15) | 965 (7) | 5,232 (37) | 4,525 (32) | 3,349 (24) |
| 10th (most advantaged/least disadvantage) | 10,701 (11) | 379 (4) | 3,494 (33) | 3,449 (32) | 3,379 (32) |
| **Care and support needs:** |  |  |  |  |  |
| **Physical considerations: activities-of-daily-living** |  |  |  |  |  |
| ***Mobility*** |  |  |  |  |  |
| ***Walking*** |  |  |  |  |  |
| No assistance | 38,777 (41) | 3,755 (10) | 20,358 (53) | 10,470 (27) | 4,194 (11) |
| Some assistance | 54,190 (57) | 1,122 (2) | 15,256 (28) | 21,325 (39) | 16,487 (30) |
| High level of assistance/unable | 2,008 (2) | 6 (0) | 109 (5) | 236 (12) | 1,657 (83) |
| ***Transfers ^%^*** |  |  |  |  |  |
| No assistance | 56,851 (60) | 4,373 (8) | 27,295 (48) | 17,375 (31) | 7,808 (14) |
| Some assistance | 37,052 (39) | 508 (1) | 8,393 (23) | 14,582 (39) | 13,569 (37) |
| High level of assistance/unable | 1,072 (1) | 2 (0) | 35 (3) | 74 (7) | 961 (90) |
| ***Personal care*** |  |  |  |  |  |
| ***Showering/bathing*** |  |  |  |  |  |
| No assistance | 44,977 (47) | 4,391 (10) | 26,261 (58) | 11,363 (25) | 2,962 (7) |
| Some assistance | 46,927 (49) | 489 (1) | 9,348 (20) | 20,301 (43) | 16,789 (36) |
| High level of assistance/unable | 3,071 (3) | 3 (0) | 114 (4) | 367 (12) | 2,587 (84) |
| ***Dressing*** |  |  |  |  |  |
| No assistance | 53,836 (57) | 4,434 (8) | 28,704 (53) | 15,996 (30) | 4,702 (9) |
| Some assistance | 38,654 (41) | 448 (1) | 6,950 (18) | 15,806 (41) | 15,450 (40) |
| High level of assistance/unable | 2,485 (3) | 1 (0) | 69 (3) | 229 (9) | 2,186 (88) |
| ***Toileting (bladder)*** |  |  |  |  |  |
| No assistance | 67,413 (71) | 4,540 (7) | 30,777 (46) | 22,861 (34) | 9,235 (14) |
| Some assistance | 25,667 (27) | 342 (1) | 4,871 (19) | 8,963 (35) | 11,491 (45) |
| High level of assistance/unable | 1,895 (2) | 1 (0) | 75 (4) | 207 (11) | 1,612 (85) |
| ***Toileting (bowel) ^j^*** |  |  |  |  |  |
| No assistance | 75,795 (80) | 4,740 (6) | 33,131 (44) | 26,227 (35) | 11,697 (15) |
| Some assistance | 17,824 (19) | 142 (1) | 2,540 (14) | 5,708 (32) | 9,434 (53) |
| High level of assistance/unable | 1,354 (1) | 1 (0) | 50 (4) | 96 (7) | 1,207 (89) |
| ***Medication management*** |  |  |  |  |  |
| No assistance | 41,162 (43) | 4,087 (10) | 22,834 (56) | 11,354 (28) | 2,887 (7) |
| Some assistance | 49,862 (53) | 788 (2) | 12,615 (25) | 19,746 (40) | 16,713 (34) |
| High level of assistance/unable | 3,951 (4) | 8 (0) | 274 (7) | 931 (24) | 2,738 (69) |
| ***Eating/feeding*** |  |  |  |  |  |
| No assistance | 78,170 (82) | 4,806 (6) | 34,090 (44) | 26,922 (34) | 12,352 (16) |
| Some assistance | 16,432 (17) | 77 (1) | 1,618 (10) | 5,087 (31) | 9,650 (59) |
| High level of assistance/unable | 373 (0) | 0 (0) | 15 (4) | 22 (6) | 336 (90) |
| ***Domestic tasks*** |  |  |  |  |  |
| ***Meal preparation*** |  |  |  |  |  |
| No assistance | 18,155 (19) | 2,685 (15) | 11,449 (63) | 3,314 (18) | 707 (4) |
| Some assistance | 56,553 (60) | 2,086 (4) | 22,154 (39) | 22,262 (39) | 10,051 (18) |
| High level of assistance/unable | 20,267 (21) | 112 (1) | 2,120 (11) | 6,455 (32) | 11,580 (57) |
| ***Housework ^k^*** |  |  |  |  |  |
| No assistance | 2,657 (3) | 474 (18) | 1,562 (59) | 461 (17) | 160 (6) |
| Some assistance | 62,588 (66) | 4,152 (7) | 29,709 (48) | 20,982 (34) | 7,745 (12) |
| High level of assistance | 29,729 (31) | 257 (1) | 4,452 (15) | 10,588 (36) | 14,432 (49) |
| ***Community access*** |  |  |  |  |  |
| ***Transport ^k^*** |  |  |  |  |  |
| No assistance | 13,178 (14) | 2,623 (20) | 8,214 (62) | 1,965 (15) | 376 (3) |
| Some assistance | 76,222 (80) | 2,225 (3) | 26,870 (35) | 28,680 (38) | 18,447 (24) |
| High level of assistance/unable | 5,574 (6) | 35 () | 639 (12) | 1,386 (25) | 3,514 (63) |
| ***Shopping*** |  |  |  |  |  |
| No assistance | 9,809 (10) | 2,219 (23) | 6,018 (61) | 1,307 (13) | 265 (3) |
| Some assistance | 57,843 (61) | 2,476 (4) | 26,030 (45) | 20,934 (36) | 8,403 (15) |
| High level of assistance/unable | 27,323 (29) | 188 (1) | 3,675 (14) | 9,790 (36) | 13,670 (50) |
| ***Handling money/paying bills*** |  |  |  |  |  |
| No assistance | 30,688 (32) | 3,406 (11) | 17,029 (56) | 7,995 (26) | 2,258 (7) |
| Some assistance | 47,310 (50) | 1,411 (3) | 16,844 (36) | 18,478 (39) | 10,577 (22) |
| High level of assistance/unable | 16,977 (18) | 66 (0) | 1,850 (11) | 5,558 (33) | 9,503 (56) |
| ***Home maintenance concerns ^l^*** | 52,239 (55) | 2,496 (5) | 19,250 (37) | 17,674 (34) | 12,819 (25) |
| **Physical considerations: other care concerns** |  |  |  |  |  |
| Poor vision ^^^ | 61,077 (64) | 2,834 (5) | 22,760 (37) | 21,142 (35) | 14,341 (24) |
| Blindness ^^^ | 3,093 (3) | 68 (2) | 858 (28) | 1,172 (38) | 995 (32) |
| Poor hearing ^^^ | 45,377 (48) | 2,018 (5) | 16,506 (36) | 15,640 (35) | 11,213 (25) |
| Deafness ^^^ | 4,545 (5) | 200 (4) | 1,485 (33) | 1,555 (34) | 1,305 (29) |
| Speech issues ^^^ | 7,221 (8) | 132 (2) | 1,314 (18) | 2,475 (34) | 3,300 (46) |
| Swallowing problems ^a^ | 12,771 (14) | 307 (2) | 3,282 (26) | 4,481 (35) | 4,701 (37) |
| Communication difficulties ^l^ | 35,415 (37) | 772 (2) | 9,075 (26) | 12,683 (36) | 12,885 (36) |
| Oral health concerns ^a^ | 20,770 (22) | 826 (4) | 6,961 (34) | 7,186 (35) | 5,797 (28) |
| Food, fluid and/or weight concerns ^j^ | 38,921 (41) | 1,059 (3) | 11,692 (30) | 14,156 (36) | 12,014 (31) |
| Major skin conditions ^a^ | 33,387 (35) | 1,211 (4) | 10,879 (33) | 11,570 (35) | 9,727 (29) |
| Has difficulty sleeping ^a^ | 51,944 (55) | 2,364 (5) | 19,028 (37) | 17,659 (34) | 12,893 (25) |
| Falls/slips/trips in last 12 months ^k^ | 56,066 (59) | 1,962 (4) | 18,687 (33) | 20,110 (36) | 15,307 (27) |
| Has inadequate physical activity ^l^ | 70,841 (75) | 3,020 (4) | 24,780 (35) | 24,764 (35) | 18,277 (26) |
| Experienced bodily pain in last 4 weeks ^k^ | 71,261 (75) | 3,751 (5) | 27,514 (39) | 24,216 (34) | 15,780 (22) |
| **Cognitive/behavioural/psychological considerations:** |  |  |  |  |  |
| ***Decision making assistance*** |  |  |  |  |  |
| Health/lifestyle decisions ^#^ | 38,264 (40) | 877 (2) | 10,562 (28) | 13,636 (36) | 13,189 (35) |
| Financial decisions ^#^ | 63,906 (67) | 2,244 (4) | 21,319 (33) | 22,384 (35) | 17,959 (28) |
| ***Specific cognitive care concerns*** |  |  |  |  |  |
| ***Short term memory loss ^j^*** |  |  |  |  |  |
| Never | 22,872 (24) | 2,222 (10) | 11,181 (49) | 6,518 (29) | 2,951 (13) |
| Occasionally | 44,076 (46) | 2,317 (5) | 18,796 (43) | 15,253 (35) | 7,710 (18) |
| Regularly | 21,806 (23) | 309 (1) | 5,117 (24) | 8,484 (39) | 7,896 (36) |
| Always | 5,588 (6) | 23 (0) | 492 (9) | 1,608 (29) | 3,465 (62) |
| Unable to determine | 631 (1) | 11 (2) | 137 (22) | 168 (27) | 315 (50) |
| ***Long term memory loss ^j^*** |  |  |  |  |  |
| Never | 67,373 (71) | 4,421 (7) | 29,556 (44) | 22,257 (33) | 11,139 (17) |
| Occasionally | 19,862 (21) | 399 (2) | 5,228 (26) | 7,488 (38) | 6,747 (34) |
| Regularly | 5,088 (5) | 34 (1) | 555 (11) | 1,545 (30) | 2,954 (58) |
| Always | 778 (1) | 5 (1) | 33 (4) | 118 (15) | 622 (80) |
| Unable to determine | 1,872 (2) | 23 (1) | 351 (19) | 623 (33) | 875 (47) |
| ***Disorientation - time ^k^*** |  |  |  |  |  |
| Never | 66,968 (71) | 4,483 (7) | 29,983 (45) | 21,863 (33) | 10,639 (16) |
| Occasionally | 17,694 (19) | 329 (2) | 4,542 (26) | 6,906 (39) | 5,917 (33) |
| Regularly | 7,349 (8) | 42 (1) | 820 (11) | 2,462 (34) | 4,025 (55) |
| Always | 1,496 (2) | 3 (0) | 78 (5) | 343 (23) | 1,072 (72) |
| Unable to determine | 1,467 (2) | 25 (2) | 300 (21) | 457 (31) | 685 (47) |
| ***Disorientation - place ^k^*** |  |  |  |  |  |
| Never | 79,580 (84) | 4,765 (6) | 33,352 (42) | 26,814 (34) | 14,649 (18) |
| Occasionally | 10,101 (11) | 89 (1) | 1,860 (18) | 3,770 (37) | 4,382 (43) |
| Regularly | 3,205 (3) | 10 (0) | 202 (6) | 858 (27) | 2,135 (67) |
| Always | 542 (1) | 0 (0) | 19 (4) | 89 (16) | 434 (80) |
| Unable to determine | 1,546 (2) | 18 (1) | 290 (19) | 500 (32) | 738 (48) |
| ***Disorientation - person ^k^*** |  |  |  |  |  |
| Never | 82,027 (86) | 4,757 (6) | 33,547 (41) | 27,859 (34) | 15,864 (19) |
| Occasionally | 8,945 (9) | 95 (1) | 1,741 (20) | 3,119 (35) | 3,990 (45) |
| Regularly | 2,211 (2) | 11 (1) | 151 (7) | 540 (24) | 1,509 (68) |
| Always | 283 (0) | 0 (0) | 12 (4) | 40 (14) | 231 (82) |
| Unable to determine | 1,508 (2) | 19 (1) | 272 (18) | 473 (31) | 744 (49) |
| ***Hallucinations ^k^*** |  |  |  |  |  |
| Never | 83,274 (88) | 4,743 (6) | 33,533 (40) | 28,042 (34) | 16,956 (20) |
| Occasionally | 5,766 (6) | 69 (1.) | 1,063 (18) | 2,057 (36) | 2,577 (45) |
| Regularly | 1,655 (2) | 6 (0) | 185 (11) | 460 (28) | 1,004 (61) |
| Always | 109 (0) | 2 (2) | 6 (6) | 24 (22) | 77 (71) |
| Unable to determine | 4,170 (4) | 62 (2) | 936 (23) | 1,448 (35) | 1,724 (41) |
| ***Specific behavioural care concerns*** |  |  |  |  |  |
| ***Impaired judgement ^j^*** |  |  |  |  |  |
| Never | 68,483 (72) | 4,528 (7) | 30,267 (44) | 22,381 (33) | 11,307 (17) |
| Occasionally | 16,807 (18) | 212 (1) | 3,766 (22) | 6,641 (40) | 6,188 (37) |
| Regularly | 4,886 (5) | 18 (0) | 458 (9) | 1,445 (30) | 2,965 (61) |
| Always | 634 (1) | 1 (0) | 29 (5) | 94 (15) | 510 (80) |
| Unable to determine | 4,163 (4) | 123 (3) | 1,203 (29) | 1,470 (35) | 1,367 (33) |
| ***Risky behaviour ^k^*** |  |  |  |  |  |
| Never | 76,400 (80) | 4,631 (6) | 31,852 (42) | 25,401 (33) | 14,516 (19) |
| Occasionally | 13,237 (14) | 169 (1) | 2,806 (21) | 4,853 (37) | 5,409 (41) |
| Regularly | 2,747 (3) | 19 (1) | 385 (14) | 842 (31) | 1,501 (55) |
| Always | 210 (0) | 0 (0) | 16 (8) | 31 (15) | 163 (78) |
| Unable to determine | 2,380 (3) | 63 (3) | 664 (28) | 904 (38) | 749 (32) |
| ***Verbal aggression **** |  |  |  |  |  |
| Never | 82,920 (87) | 4,659 (6) | 33,156 (40) | 27,627 (33) | 17,478 (21) |
| Occasionally | 9,211 (10) | 161 (2) | 2,047 (22) | 3,400 (37) | 3,603 (39) |
| Regularly | 1,778 (2) | 24 (1) | 204 (12) | 628 (35) | 922 (52) |
| Always | 51 (0) | 0 (0) | 3 (6) | 13 (26) | 35 (69) |
| Unable to determine | 1,013 (1) | 38 (4) | 313 (31) | 363 (36) | 299 (30) |
| ***Physical aggression ^k^*** |  |  |  |  |  |
| Never | 92,293 (97) | 4,835 (5) | 35,274 (38) | 31,176 (34) | 21,008 (23) |
| Occasionally | 1,675 (2) | 16 (1) | 224 (13) | 498 (30) | 937 (56) |
| Regularly | 133 (0) | 1 (1) | 10 (8) | 23 (17) | 99 (74) |
| Always | 4 (0) | 0 (0) | 0 (0) | 0 (0) | 4 (100) |
| Unable to determine | 869 (1) | 30 (4) | 215 (25) | 334 (38) | 290 (33) |
| ***Agitation ^j^*** |  |  |  |  |  |
| Never | 73,256 (77) | 4,347 (6) | 30,305 (41) | 24,280 (33) | 14,324 (20) |
| Occasionally | 17,105 (18) | 440 (3) | 4,469 (26) | 6,226 (36) | 5,970 (35) |
| Regularly | 3,147 (3) | 57 (2) | 525 (17) | 1,024 (33) | 1,541 (49) |
| Always | 110 (0) | 2 (2) | 16 (15) | 33 (30) | 59 (54) |
| Unable to determine | 1,355 (1) | 36 (3) | 408 (30) | 468 (35) | 443 (33) |
| ***Apathy **** |  |  |  |  |  |
| Never | 71,812 (76) | 4,351 (6) | 29,614 (41) | 23,722 (33) | 14,125 (20) |
| Occasionally | 12,894 (14) | 344 (3) | 3,802 (30) | 4,794 (37) | 3,954 (31) |
| Regularly | 4,499 (5) | 50 (1) | 881 (20) | 1,676 (37) | 1,892 (42) |
| Always | 448 (1) | 2 (1) | 57 (13) | 122 (27) | 267 (60) |
| Unable to determine | 5,320 (6) | 135 (3) | 1,369 (26) | 1,717 (32) | 2,099 (40) |
| ***Wandering ^j^*** |  |  |  |  |  |
| Never | 89,597 (94) | 4,849 (5) | 35,097 (39) | 30,392 (34) | 19,259 (22) |
| Occasionally | 3,766 (4) | 16 (0) | 405 (11) | 1,222 (33) | 2,123 (56) |
| Regularly | 819 (1) | 2 (0) | 56 (7) | 171 (21) | 590 (72) |
| Always | 68 (0) | 0 (0) | 5 (7) | 9 (13) | 54 (79) |
| Unable to determine | 723 (1) | 15 (2) | 160 (22) | 237 (33) | 311 (43) |
| ***Resistive behaviour ^k^*** |  |  |  |  |  |
| Never | 76,664 (81) | 4,584 (6) | 31,692 (41) | 25,425 (33) | 14,963 (20) |
| Occasionally | 13,184 (14) | 218 (2) | 3,098 (24) | 4,864 (37) | 5,004 (38) |
| Regularly | 3,770 (4) | 31 (1) | 539 (14) | 1,241 (33) | 1,959 (52) |
| Always | 173 (0) | 3 (2) | 15 (9) | 50 (29) | 105 (61) |
| Unable to determine | 1,183 (1) | 46 (4) | 379 (32) | 451 (38) | 307 (26) |
| ***Confusion ^j^*** |  |  |  |  |  |
| Never | 61,949 (65) | 4,469 (7) | 28,520 (46) | 19,823 (32) | 9,137 (15) |
| Occasionally | 22,525 (24) | 352 (2) | 5,931 (26) | 8,935 (40) | 7,307 (32) |
| Regularly | 7,924 (8) | 22 (0) | 811 (10) | 2,555 (32) | 4,536 (57) |
| Always | 1,114 (1) | 0 (0) | 46 (4) | 219 (20) | 849 (76) |
| Unable to determine | 1,461 (2) | 39 (3) | 415 (28) | 499 (34) | 508 (35) |
| ***Specific psychological care concerns*** |  |  |  |  |  |
| ***Insomnia ^j^*** |  |  |  |  |  |
| Never | 31,916 (34) | 1,912 (6) | 12,596 (40) | 10,685 (34) | 6,723 (21) |
| Occasionally | 30,039 (32) | 1,489 (5) | 11,336 (38) | 10,138 (34) | 7,076 (24) |
| Regularly | 27,651 (29) | 1,244 (5) | 9,959 (36) | 9,530 (35) | 6,918 (25) |
| Always | 4,875 (5) | 227 (5) | 1,738 (36) | 1,541 (32) | 1,369 (28) |
| Unable to determine | 492 (1) | 10 (2) | 94 (19) | 137 (28) | 251 (51) |
| ***Depression ^j^*** |  |  |  |  |  |
| Never | 39,749 (42) | 2,953 (7) | 16,934 (43) | 12,455 (31) | 7,407 (19) |
| Occasionally | 36,679 (39) | 1,482 (4) | 13,465 (37) | 12,889 (35) | 8,843 (24) |
| Regularly | 12,413 (13) | 335 (3) | 3,827 (31) | 4,732 (38) | 3,519 (28) |
| Always | 1,114 (1) | 27 (2) | 302 (27) | 411 (37) | 374 (34) |
| Unable to determine | 5,018 (5) | 85 (2) | 1,195 (24) | 1,544 (31) | 2,194 (44) |
| ***Anxiety ^j^*** |  |  |  |  |  |
| Never | 36,576 (39) | 2,524 (7) | 15,295 (42) | 11,714 (32) | 7,043 (19) |
| Occasionally | 38,744 (41) | 1,774 (5) | 14,375 (37) | 13,347 (35) | 9,248 (24) |
| Regularly | 14,950 (16) | 476 (3) | 4,852 (33) | 5,458 (37) | 4,164 (28) |
| Always | 1,440 (2) | 52 (4) | 409 (28) | 510 (35) | 469 (33) |
| Unable to determine | 3,263 (3) | 56 (2) | 792 (24) | 1,002 (31) | 1,413 (43) |
| ***Loneliness ^m^*** |  |  |  |  |  |
| Never | 51,430 (54) | 3,508 (7) | 20,833 (41) | 16,344 (32) | 10,745 (21) |
| Occasionally | 30,811 (32) | 1,103 (4) | 11,440 (37) | 11,183 (36) | 7,085 (23) |
| Regularly | 7,821 (8) | 192 (3) | 2,347 (30) | 3,075 (39) | 2,207 (28) |
| Always | 616 (1) | 23 (4) | 157 (26) | 213 (35) | 223 (36) |
| Unable to determine | 4,257 (5) | 56 (1) | 930 (22) | 1,203 (28) | 2,068 (49) |
| ***Social isolation ^n^*** |  |  |  |  |  |
| Never | 44,845 (47) | 3,358 (8) | 18,820 (42) | 13,693 (31) | 8,974 (20) |
| Occasionally | 34,234 (36) | 1,212 (4) | 12,683 (37) | 12,419 (36) | 7,920 (23) |
| Regularly | 12,161 (13) | 262 (2) | 3,378 (28) | 4,794 (39) | 3,727 (31) |
| Always | 1,100 (1) | 20 (2) | 211 (19) | 395 (36) | 474 (43) |
| Unable to determine | 2,554 (3) | 29 (1) | 615 (24) | 705 (28) | 1,205 (47) |
| **Psychosocial considerations:** |  |  |  |  |  |
| **Social support situation/carer status** |  |  |  |  |  |
| Has a carer ^j^ | 76,870 (81) | 2,687 (4) | 26,059 (34) | 27,526 (36) | 20,598 (27) |
| Is themselves a carer ^j^ | 15,210 (16) | 2,084 (14) | 8,827 (58) | 3,349 (22) | 950 (6) |
| **Vulnerability factors ^@^** |  |  |  |  |  |
| Socially isolated | 57,218 (60) | 1,796 (3) | 20,375 (36) | 20,646 (36) | 14,401 (25) |
| Culturally & linguistically diverse | 34,279 (36) | 1,453 (4) | 12,253 (36) | 11,561 (34) | 9,012 (26) |
| Carer sustainability concerns | 20,236 (21) | 442 (2) | 5,481 (27) | 7,175 (36) | 7,138 (35) |
| War veterans | 1,032 (1) | 41 (4) | 323 (31) | 386 (37) | 282 (27) |
| Aboriginal & Torres Strait Islanders | 684 (1) | 29 (4) | 260 (38) | 249 (36) | 146 (21) |
| Asylum seeker or refugee | 398 (0) | 10 (3) | 113 (28) | 147 (37) | 128 (32) |
| Gender diverse | 127 (0) | 5 (4) | 44 (35) | 33 (26) | 45 (35) |
| **Muti-vulnerabilities ^@^** |  |  |  |  |  |
| 0 | 19,873 (21) | 2,017 (10) | 8,647 (44) | 5,786 (29) | 3,423 (17) |
| 1 | 42,529 (45) | 2,041 (5) | 16,739 (39) | 14,626 (34) | 9,123 (22) |
| 2 | 26,430 (28) | 743 (3) | 8,933 (34) | 9,347 (35) | 7,407 (28) |
| 3+ | 6,143 (7) | 82 (1) | 1,404 (23) | 2,272 (37) | 2,385 (39) |
| **Complexity indicators ^@^** |  |  |  |  |  |
| Significant cognitive changes | 27,350 (29) | 213 (1) | 4,734 (17) | 10,194 (37) | 12,209 (45) |
| Self-neglect | 16,168 (17) | 148 (1) | 2,295 (14) | 5,856 (36) | 7,869 (49) |
| Emotional or mental health issues | 10,182 (11) | 173 (2) | 2,577 (25) | 3,981 (39) | 3,451 (34) |
| Financial disadvantage | 5,319 (6) | 136 (3) | 1,566 (29) | 2,000 (38) | 1,617 (30) |
| Inadequate housing | 1,649 (2) | 53 (3) | 526 (32) | 628 (38) | 442 (27) |
| Risk of, suspected or confirmed abuse | 1,555 (2) | 37 (2) | 427 (28) | 602 (39) | 489 (32) |
| Drug & alcohol use likely to cause harm to self or others | 1,245 (1) | 22 (2) | 346 (28) | 502 (40) | 375 (30) |
| History of institutionalisation | 742 (1) | 18 (2) | 182 (25) | 281 (38) | 261 (35) |
| **Multi-complexities ^@^** |  |  |  |  |  |
| 0 | 53,314 (56) | 4,245 (8) | 26,401 (50) | 16,167 (30) | 6,501 (12) |
| 1 | 25,312 (27) | 511 (2) | 6,854 (27) | 9,942 (39) | 8,005 (32) |
| 2 | 11,819 (12) | 98 (1) | 1,857 (16) | 4,274 (36) | 5,590 (47) |
| 3+ | 4,530 (5) | 29 (1) | 611 (14) | 1,648 (36) | 2,242 (50) |
| **Safety concerns** |  |  |  |  |  |
| Home safety concerns ^j^ | 28,674 (30) | 1,170 (4) | 10,575 (37) | 10,033 (35) | 6,896 (24) |
| Personal safety concerns ^j^ | 9,192 (10) | 172 (2) | 2,380 (26) | 3,300 (36) | 3,340 (36) |
| **Health concerns and healthcare use:** |  |  |  |  |  |
| **Health concerns:** |  |  |  |  |  |
| **Chronic diseases** |  |  |  |  |  |
| Musculoskeletal system diseases (including arthritis) | 54,841 (58) | 3,083 (6) | 22,382 (41) | 18,366 (34) | 11,010 (20) |
| Heart disease (includes IHD/past MI) | 30,253 (32) | 1,357 (5) | 11,255 (37) | 10,568 (35) | 7,073 (23) |
| Mental health issues | 25,630 (27) | 933 (4) | 8,917 (35) | 9,397 (37) | 6,383 (25) |
| Diabetes (includes T1DM & T2DM) | 24,854 (26) | 1,025 (4) | 8,839 (36) | 8,604 (35) | 6,386 (26) |
| Chronic lower respiratory diseases | 20,645 (22) | 908 (4) | 7,653 (37) | 7,486 (36) | 4,598 (22) |
| Eye diseases (including poor vision) | 19,421 (20) | 957 (5) | 7,478 (39) | 6,731 (35) | 4,255 (22) |
| Osteoporosis | 16,890 (18) | 794 (5) | 6,389 (38) | 5,716 (34) | 3,991 (24) |
| Diagnosed dementia (all types) | 11,438 (12) | 100 (1) | 1,970 (17) | 3,946 (35) | 5,422 (47) |
| Ear diseases (including hearing loss) | 10,539 (11) | 487 (5) | 4,052 (39) | 3,597 (34) | 2,403 (23) |
| CCF | 6,123 (6) | 158 (3) | 1,750 (29) | 2,321 (38) | 1,894 (31) |
| Parkinson's Disease | 4,618 (5) | 77 (2) | 1,010 (22) | 1,711 (37) | 1,820 (39) |
| Nutritional disorders (includes malnutrition) | 3,915 (4) | 113 (3) | 1,233 (32) | 1,385 (35) | 1,184 (30) |
| **Short term health conditions** |  |  |  |  |  |
| Delirium | 1,015 (1) | 10 (1) | 174 (17) | 338 (33) | 493 (49) |
| **Other health related signs & symptoms** |  |  |  |  |  |
| Pain | 25,120 (26) | 1,221 (5) | 10,337 (41) | 8,707 (35) | 4,855 (19) |
| Cognitive impairment (without recorded diagnosis of dementia) | 13,450 (14) | 238 (2) | 3,936 (27) | 5,661 (38) | 5,007 (34) |
| Falls | 13,931 (15) | 237 (2) | 3,974 (29) | 5,298 (38) | 4,422 (32) |
| Abnormal gait/mobility | 12,664 (13) | 208 (2) | 3,558 (28) | 4,889 (39) | 4,009 (32) |
| Incontinence (urinary or bowel) | 9,472 (10) | 198 (2) | 2,544 (27) | 3,283 (35) | 3,447 (36) |
| Vertigo and other dizziness | 6,605 (7) | 360 (6) | 2,880 (44) | 2,239 (34) | 1,126 (17) |
| **Multi-morbidity** |  |  |  |  |  |
| 0 | 18 (0) | 3 (17) | 12 (67) | 2 (11) | 1 (6) |
| 1-2 | 5,691 (6) | 540 (10) | 2,322 (41) | 1,681 (30) | 1,148 (20) |
| 3-4 | 18,735 (20) | 1,427 (8) | 7,830 (42) | 5,672 (30) | 3,806 (20) |
| 5-6 | 29,131 (31) | 1,603 (6) | 11,607 (40) | 9,779 (34) | 6,142 (21) |
| 7-8 | 25,763 (27) | 948 (4) | 9,223 (36) | 9,065 (35) | 6,527 (25) |
| 9-10 | 15,637 (17) | 362 (2) | 4,729 (30) | 5,832 (37) | 4,714 (30) |
| **Healthcare use:** |  |  |  |  |  |
| Sees a GP regularly ^j^ | 90,960 (96) | 4,678 (5) | 34,228 (38) | 30,777 (34) | 21,277 (23) |
| Using other clinical services ^j^ | 75,749 (80) | 3,671 (5) | 27,721 (37) | 25,965 (34) | 18,392 (24) |
| Health literacy support required | 45,282 (48) | 1,005 (2) | 12,307 (27) | 16,597 (37) | 15,373 (34) |
| Taking medication ^o^ | 93,551 (99) | 4,781 (5) | 35,204 (38) | 31,608 (34) | 21,958 (24) |
| *Hospitalisations ^j^:* |  |  |  |  |  |
| In past 3 months | 38,639 (41) | 1,249 (3) | 12,725 (33) | 14,022 (36) | 10,643 (28) |
| Currently in hospital | 2,667 (3) | 24 (1) | 642 (24) | 823 (31) | 1,178 (44) |

Missing data:
a: 3 (<0.001%); b: 1475 (1.6%); c: 1952 (2.1%); d: 1858 (2%); e: 256 (0.3%); f: 91 (0.1%); g: 529 (0.6%); h: 45 (0.1%); i: 50 (0.1%); j: 2 (<0.001%); k: 1 (<0.001%); l: 4 (<0.001%); m: 40 (<0.001%); n: 81 (<0.1%); o: 31 (<0.001%)

^*^ The dataset predominantly consists of individuals aged 65 years old and over which is a key eligibility criterion for Comprehensive Assessment. However, people less than 65 years old can also be assessed for eligibility for a HCP in certain and specific circumstances. From age 50: Aboriginal and Torres Strait Islanders; individuals who are prematurely aged (45 years for Aboriginal and Torres Strait Islanders) who are ‘on a low income and homeless or at risk of homelessness or living with hoarding behaviour or in a squalid environment’. Other younger people (less than age 65) with care needs can also be considered for assessment once it has been established that there is no other better system to meet their needs e.g. National Disability Insurance Scheme^2^

^%^ Transfers refers to moving about in/getting in and out of bed, as well as getting on/off chairs and the toilet, and getting in/out of the shower/car

^^^ A response is recorded for vision/blindness, hearing/deafness & speech only when this concern is applicable.

^#^ A response is recorded for specific health/lifestyle & financial decision-making assistance only when applicable.

^@^ A response is recorded for vulnerability factors & complexity indicators only recorded where applicable & >1 factor/indicator may apply per person.

Abbreviations: IRSAD = Index of Relative Advantage and Disadvantage; ADLs = activities of daily living; IHD = ischaemic heart disease, MI = myocardial infarction, T2DM = type 2 diabetes mellitus, T1DM = type 1 diabetes mellitus, COAD = chronic obstructive airways disease, COPD = chronic obstructive pulmonary disease, CCF = congestive cardiac failure, NOS = not otherwise specified, NEC = not elsewhere classified.

1. Australian Bureau of Statistics. 2033.0.55.001 - Census of Population and Housing: Socio-Economic Indexes for Areas (SEIFA), Australia, 2016. Updated 21 March 2023. Accessed 12 September, 2024. <https://www.abs.gov.au/ausstats/abs@.nsf/Lookup/by%20Subject/2033.0.55.001~2016~Main%20Features~IRSAD~20>

2. My Aged Care. *My Aged Care assessment manual - for Regional Assessment Services and Aged Care Assessment Teams (version 5.0 - 25 March 2024)*. 2024. <https://www.health.gov.au/sites/default/files/2024-06/my-aged-care-assessment-manual_0.pdf>
